# Biocatalytic Generation of *o*-Quinone Imines in the Synthesis of 1,4-Benzoxazines and Its Comparative Green Chemistry Metrics

**DOI:** 10.1021/acssuschemeng.3c06758

**Published:** 2024-02-02

**Authors:** Maryam Tehami, Hasan Tanvir Imam, Iskandar Abdullah, Joseph Hosford, Xiao Juie Wong, Noorsaadah Abd. Rahman, Lu Shin Wong

**Affiliations:** † Manchester Institute of Biotechnology, University of Manchester, 131 Princess Street, M1 7DN Manchester, United Kingdom; ‡ Department of Chemistry, University of Manchester, Oxford Road, M13 9PL Manchester, United Kingdom; § Department of Chemistry, Faculty of Science, Universiti Malaya, 50603 Kuala Lumpur, Malaysia; ∥ Institute for Advanced Studies, Universiti Malaya, 50603 Kuala Lumpur, Malaysia

**Keywords:** peroxidase, horseradish peroxidase, Diels−Alder reaction, inverse electron demand Diels−Alder, E-factor, reaction mass efficiency, tandem reaction

## Abstract

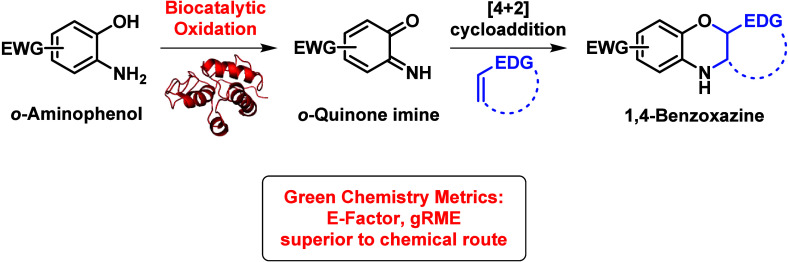

1,4-Benzoxazines are important motifs in many pharmaceuticals and can be formed by a reaction sequence involving the oxidation of *o*-aminophenols to their corresponding quinone imine followed by an *in situ* inverse electron demand Diels–Alder (IEDDA) cycloaddition with a suitable dienophile. Reported herein is the development of a reaction sequence that employs horseradish peroxidase to catalyze the oxidation of the aminophenols prior to the IEDDA as a more sustainable alternative to the use of conventional stoichiometric oxidants. The synthesis of 10 example benzoxazines is demonstrated in this “one-pot, two-step” procedure with yields between 42% and 92%. The green chemistry metrics, including the E-factor and generalized reaction mass efficiency, for this biocatalytic reaction were compared against the conventional chemical approach. It was found that the reported biocatalytic route was approximately twice as green by these measures.

## Abstract 2

A peroxidase-catalyzed oxidation incorporated into an oxidation−cycloaddition reaction sequence enables a greener synthesis of benzoxazines.

## Introduction

*o*-Quinone imines are transitory and highly reactive species that are versatile intermediates in organic synthesis.^1^ They readily undergo a variety of reactions, including 1,4-conjugate additions,^2^ 1,3-oxazole formation,^3^ and [4 + 2] inverse electron demand Diels–Alder cycloadditions (IEDDA).^1,4,5^ The IEDDA reactions are of interest since they give rise to a 1,4-benzoxazine scaffold that is present in a variety of complex molecules including drug candidates, as well as approved medicines (e.g., azasetron and levofloxacin).^6−8^ In this context, IEDDA reactions are particularly useful since their concerted reaction mechanism imparts good control of both regio- and stereochemistry in comparison to stepwise reactions^9,10^ of forming benzoxazines.

**Scheme 1:**
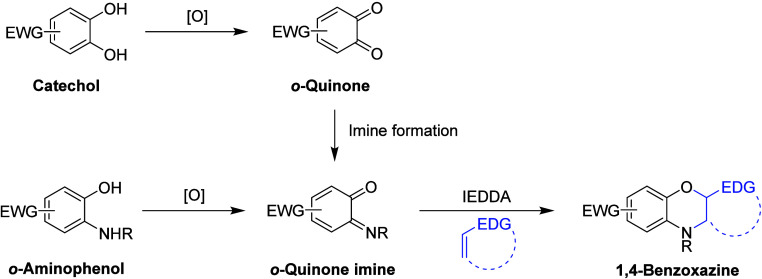
General Scheme for the Oxidative Generation of *o*-Quinone Imines, Followed by the Inverse Electron Demand Diels–Alder (IEDDA) Reaction R = H, alkyl, or aryl; EWG = electron-withdrawing group; EDG = electron-donating group.

These *o*-quinone imines can be synthetically accessed through two general routes (Scheme 1). First, they can be produced from the corresponding catechol by oxidation to its quinone,^11^ followed by condensation with an amine,^12−14^ iminophosporane,^15^ or arsinimine.^16^ However, this route from the catechol presents two drawbacks: there is a need to produce the relatively reactive *o*-quinone intermediate that is susceptible to undesirable side reactions (e.g., conjugate additions and polymerization), and the imine formation can in some cases require harsh conditions, such as elevated temperatures and the use of strong acids and bases. An alternative approach to *o*-quinone imines is through the oxidation of the corresponding *o*-aminophenol. This oxidation can be affected with a variety of stoichiometric reagents including the classical metal oxidants (e.g., chromium(VI), iron(III), and cerium(IV)),^1,17^ hypervalent iodine compounds,^18,19^ or sodium hypochlorite.^1,20^ Oxidations of *o*-aminophenol have also been reported using oxygen or hydrogen peroxide as the terminal oxidant in the presence of a transition metal as a catalyst^17,21^ and through electrochemical methods.^5,22,23^ More recently, the photochemical generation from the corresponding *o*-azidophenol has also been reported in the context of bioconjugation,^24^ though the preparation of the azidophenol starting materials is perhaps less suitable in a synthetic context.

Tandem reaction sequences whereby the generation of the *o*-quinone imine is coupled to an IEDDA reaction have also been demonstrated in a “one-pot” manner. One example demonstrated by Bodipati and Peddinti involved the *in situ* generation of *o*-benzoquinone imines from their corresponding electron deficient *o*-anminophenols by oxidation with the hypervalent iodine reagent (diacetoxyiodo)benzene (DAIB).^19^ The *o*-quinone imines subsequently reacted with a range of dienophiles *in situ* to produce a series of 1,4-benzoxazines. In another example, oxidation of the aminophenol by periodate followed by the IEDDA could be carried out under conditions that were sufficiently mild for the bioconjugation of viral capsid proteins.^25^

An alternative method that could be more desirable from a sustainability perspective is to employ enzyme-catalyzed oxidation. Here, biocatalysis has the advantages of being able to carry out reactions in aqueous conditions, under ambient temperature and pH, and with high chemoselectivity.^26^ Heme-dependent peroxidases such as horseradish peroxidase (HRP)^27^ are known to be capable of oxidizing catechols,^28^*o*-aminophenols,^29^ and *o*-diaminobenzenes^30^ to their corresponding quinoid species using readily available and nontoxic hydrogen peroxide as the terminal oxidant. HRP also has the advantages of being sustainably sourced and being commercially available. Previous work has demonstrated that HRP can be utilized in an oxidation of 4-methylcatechol that is coupled with a conjugate addition in a “one-pot” procedure.^31^ These examples suggest the possibility of using biocatalysis in a chemical synthetic context to generate quinone imines for other synthetic sequences.

This report details efforts to integrate an HRP-based biocatalytic oxidation with IEDDA into a tandem one-pot procedure. Subsequently, the sustainability of the reported method was assessed in comparison with the purely chemical approach by an analysis of the reactions’ various environmental impact metrics.

## Results and Discussion

### Experimental Design and Optimisation

**Figure 1:**
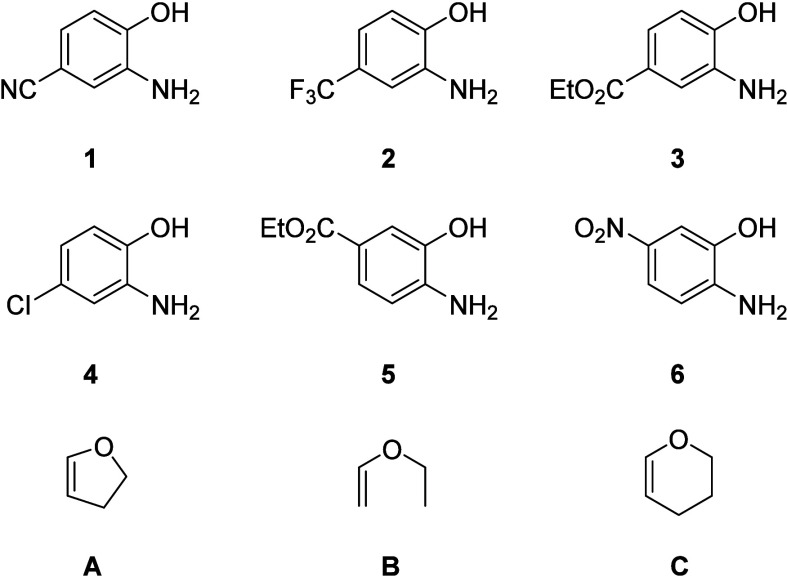
Structures of the aminophenols and dienophiles used in this study. **1** = 3-Amino-4-hydroxybenzonitrile; **2** = 2-amino-4-(trifluoromethyl)phenol; **3** = ethyl 3-amino-4-hydroxybenzoate; **4** = 2-amino-4-chlorophenol; **5** = ethyl 4-amino-3-hydroxybenzoate; **6** = 2-amino-5-nitrophenol; **A** = 2,3-dihydrofuran; **B** = ethoxyethane; and **C** = 3,4-dihydro-2*H*-pyran.

For this investigation, a selection of aminophenols **1**–**6** bearing a range of electron-withdrawing groups was used (Figure 1). 2,3-Dihydrofuran (**A**), ethoxyethene (**B**), and 3,4-dihydro-2*H*-pyran (**C**) were chosen as dienophiles as they have previously been shown to participate in IEDDA reactions (Figure 1).^32^

**Figure 2:**
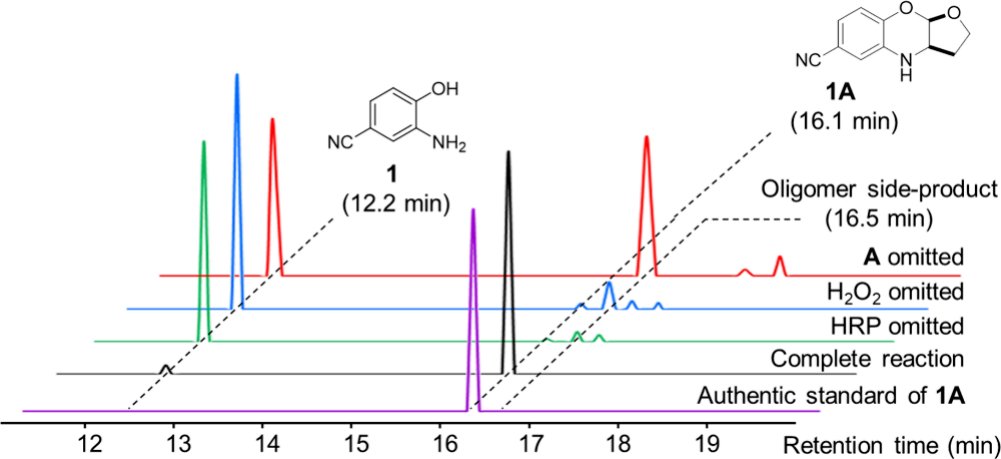
Illustrative HPLC chromatograms of the reaction mixture involving **1** and **A** with the fully constituted reaction (black) or with the omission of HRP (green), H_2_O_2_ (blue), or the dienophile **A** (red). The chromatogram for an authentic sample of **1A** is also shown (purple). The structures of **1** and *endo*-**1A** (only relative stereochemistry indicated) are shown in the inset. Reaction conditions: 1 mM **1**, 50 mM **2**, 1 mM H_2_O_2_, and 2.5 U of HRP mL^–1^ in 50 mM phosphate buffer (pH 7.2), RT, 12 h.

The aminophenol **1** and furan **A** (to give the product benzoxazine **1A**^a^) were first chosen for the initial testing since these were known to readily undergo IEDDA upon oxidation of **1**.^19^ The aminophenol, dienophile, and H_2_O_2_ were first mixed in aqueous buffer, with a minimal amount of 1,4-dioxane to solubilize the organic compounds where necessary; and the reaction commenced by the addition of HRP. Analytical scale reactions (0.3 nmol) were carried out and analyzed by LCMS (Figure 2). As controls, parallel reactions were also carried out where either the enzyme, H_2_O_2_, or the dienophile was omitted. The expected benzoxazine product was then identified by HRMS and comparison with chromatograms of authentic samples that were synthesized by conventional synthetic methods.^19^

In the fully constituted reaction, one major product was found (88% conversion by HPLC peak area) with the retention time and mass spectrum that corresponded to the expected *endo*-**1A** product. This is the same major product that is generated from the purely chemical route that used diacetoxyiodobenzene (DAIB) as the oxidant in a one-pot reaction.^19^ The reaction was found to be rapid, with most of the aminophenol being converted after 10 min (72% conversion) and the reaction reaching completion after 80 min (88% conversion, Figure S1). In the control reactions where either HRP or H_2_O_2_ were omitted, only unaltered starting materials were observed. In the control where the dienophile was omitted, a complex mixture of products together with the formation of a white precipitate were observed, which were likely the oligomers and polymers that arise from the oxidative condensation of the aminophenol.^33^

**Figure 3:**
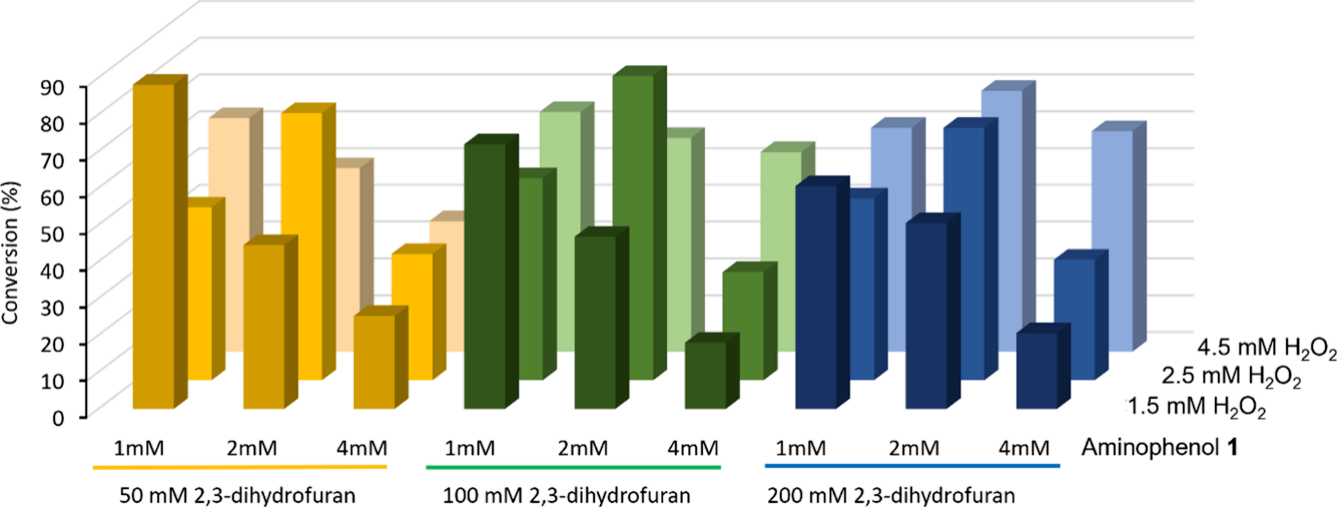
Bar chart of percentage reaction conversions (measured by the HPLC peak area of the 1,4-benzoxazine product) against varying concentrations of H_2_O_2_, 3-amino-4-hydroxybenzonitrile (**1**), and 2,3-dihydrofuran (**A**). A 0.25 U mL^–1^ concentration of HRP and a reaction time of 60 min was used in all cases.

Having demonstrated its feasibility, further reaction optimization was conducted for the formation of **1A**. When varying the amount of HRP, it was found that good conversions were obtained using 0.25 U mL^–1^ of enzyme, with little change in conversion even when increased by up to 2 orders of magnitude (to 25 U mL^–1^, Figure S2). In regard to the stoichiometry between H_2_O_2_ and the substrate (Figure 3), best results were generally obtained with 1.25 or 1.5 mol equiv of H_2_O_2_ (i.e., 2.5 mM H_2_O_2_ for 2.0 mM aminophenol, or 1.5 mM H_2_O_2_ for 1.0 mM aminophenol). This observation is consistent with previous studies showing that high concentrations of H_2_O_2_ can result in “suicide inactivation” of peroxidases due to the formation of highly reactive intermediate radicals that attack and degrade the enzyme.^34,35^ For the concentration of the dienophile, essentially similar outcomes were obtained for all of the concentrations that were tested. This result was unsurprising since the dienophiles were already used in molar excess in all cases.

A semipreparative scale reaction (0.6 mmol of aminophenol) was then repeated to enable a fuller characterization of the product. This analysis confirmed the structure of **1A** and an optical rotation measurement indicated this product was racemic. These findings were consistent with the postulated mechanism, whereby the enzyme generates the achiral quinone imine that then leaves the enzyme active site to undergo the IEDDA reaction, and thus no stereochemical induction was expected.

### Substrate Scoping and Scale-Up

Using the optimized reaction conditions, the other aminophenols **2**–**6** were subjected to the same reaction sequence with each of the dienophiles **A**–**C**. These scoping reactions were first performed at a small scale (nanomole to micromole range) and analyzed by HPLC. Any reactions that successfully generated the cycloaddition product were then repeated at a preparative scale (millimole quantities and at higher reactant concentrations) to enable compound isolation, calculation of the isolated yield, and compound characterization.

**Figure 4:**
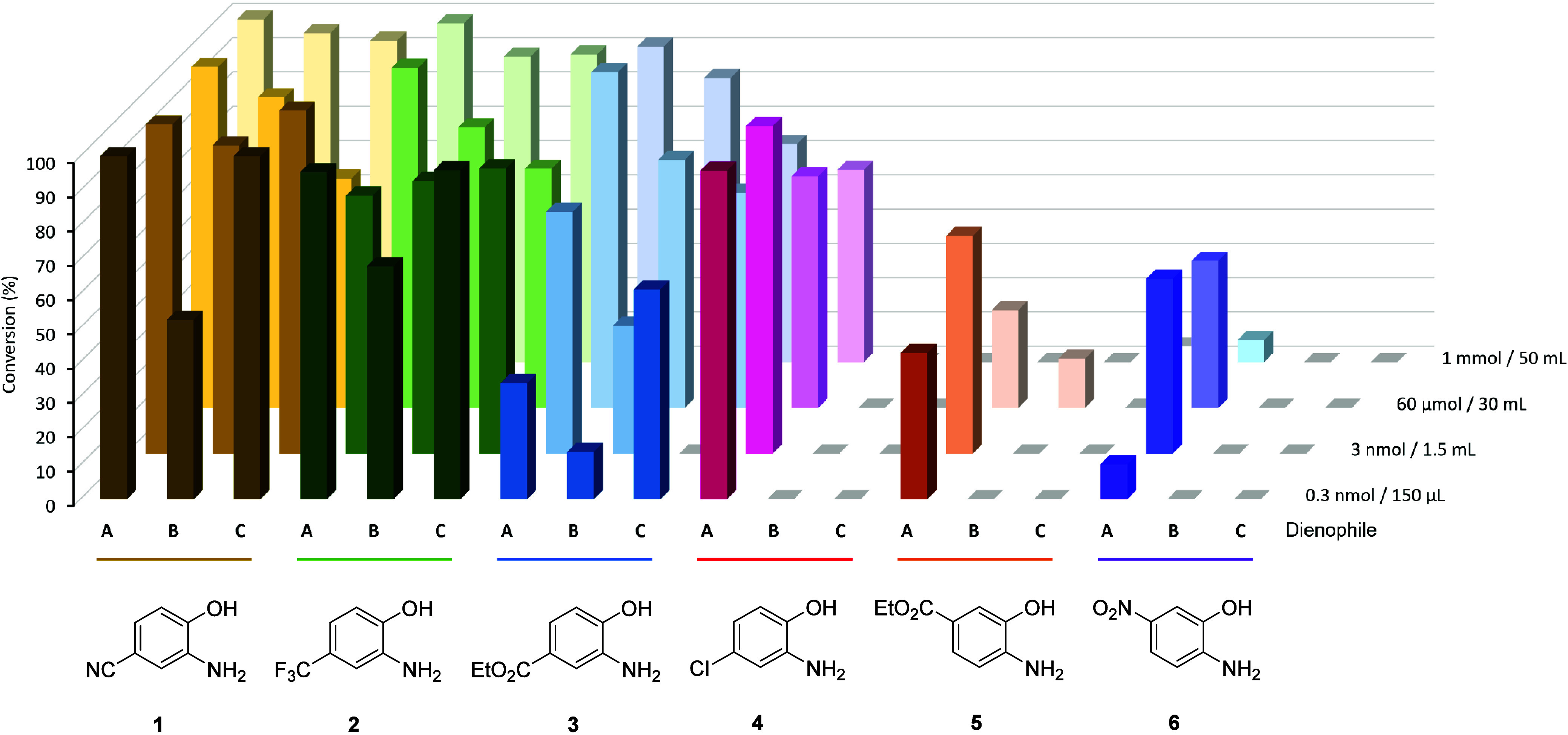
Bar chart of percentage reaction conversion of the oxidation-IEDDA sequence (measured by HPLC peak area of the 1,4-benzoxazine product) for each pair of aminophenol and dienophile reactants over a range of reaction scales (quoted as quantity of aminophenol and reaction volume). Gray squares indicate no benzoxazine was detected.

In general, it was found that aminophenols **1**–**3** that had strong EWGs *para* to the hydroxy group produced at least some corresponding benzoxazine even at the preparative scale (Figure 4 and Table S1), with isolated yields between 42–92% (Table 1). In all cases, the major product was the expected cycloadduct product bearing the same regio- and stereochemical outcome as the previously reported chemical synthesis (i.e., the same as **1A** shown in Figure 2).^19^ Aminophenol **4** bearing the chloro substitution that is a relatively weaker EWG gave the benzoxazine product only with furan **A**, with the preparative scale reaction giving a modest 51% isolated yield. When the other dienophiles **B** or **C** were used with **4**, only polymerization products were observed. These results indicated that the enzyme-catalyzed oxidation did occur but the resultant quinone imine was not able to undergo the subsequent IEDDA.

**Table 1:**
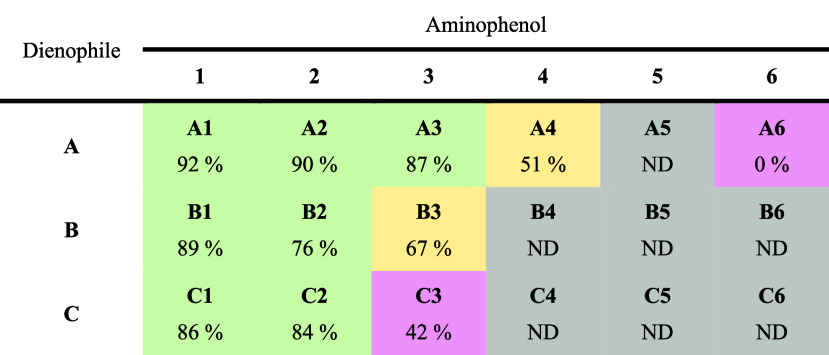
Percentage of Isolated Yields from the Preparative Scale Reaction for Each Pair of Aminophenols and Dienophiles^a^

a ND = not determined, as product conversion was not detected by HPLC. Color key: green, ≥75%; yellow, ≥50% to <75%; pink, <50%; and grey, no conversion.

The aminophenols **5** and **6**, which had the substituents *meta* relative to the hydroxy group gave low and generally unreliable conversions with **A** at the analytical scales. At the preparative scale, both aminophenols resulted in either no conversion to the product or only trace amounts (i.e., no conversion detected by HPLC or no compound isolated). Neither aminophenol gave any significant conversion with the other dienophiles. For the ethyl ester derivative **5**, this result contrasts markedly with its isomer **3**, which readily formed the benzoxazine products **3A–3C** with all three dienophiles. In all cases with **5**, this aminophenol was always observed to be consumed as the reaction progressed, which suggested that the biocatalytic oxidation of **5** was successful; thus, the lack of benzoxazine is due to the failure of the subsequent IEDDA reaction. Indeed, none of the benzoxazines **5A–5C** have been previously reported by any synthetic route.

In the case of the nitro-substituted **6**, it has been previously reported that **6A** and **6B** can be produced via DAIB oxidation of **6**.^19^ Thus, the lack of product is likely due to the inability of HRP to catalyze the oxidation of this aminophenol. Although small amounts of the desired product and polymeric material were observed in the smaller scale reactions, in all cases, the starting material **6** was observed (by HPLC) to be largely unchanged.

As a further comparison, unsubstituted 2-aminophenol **7** was also tested under the same reaction conditions with all three dienophiles (Table S1). In all cases, **7** did not produce the desired cycloadduct, and only polymerization was observed. This result is consistent with previous reports whereby HRP is indeed able to catalyze the oxidation of **7**,^29^ but the resultant quinone imine is insufficiently electron deficient to undergo subsequent IEDDA.^19^

### Green Chemistry Metrics and Comparative Analysis

To estimate the relative sustainability of this biocatalytic approach, the green metrics for the syntheses of **1A**, **1B**, **2A**, and **2B** were calculated. These representative compounds were chosen since they can be produced by “one-pot” methods involving either the HRP-catalyzed oxidation discussed above or the previously reported DAIB oxidation^19^ (i.e., both methods gave isolated yields of ≥60% for all four compounds). Here, the E-factor (E = total mass of waste/mass of product),^36,37^ the reaction mass efficiency (RME = mass of product/total mass of reactants), and generalized RME (gRME = mass of product/total mass used in a process)^38^ were calculated (Table 2); whereby the lower E-factors and higher RME or gRME values indicated lower waste generation.

**Table 2:** Calculated E-Factor, RME, gRME, and Mass of Reaction Waste for the Syntheses of **1A**, **1B**, **2A**, and **2B** Using Either DAIB or HRP/H_2_O_2_ as the Oxidant^a^

	E-factor		RME		gRME		reaction waste (g)		isolated yield (%)	
product	DAIB	HRP/H_2_O_2_	DAIB	HRP/H_2_O_2_	DAIB	HRP/H_2_O_2_	DAIB	HRP/H_2_O_2_	DAIB^19^	HRP/H_2_O_2_
**1A**	41 (1145)	24 (736)	0.024	0.098	0.00062	0.0013	6.3 (176)	4.5 (139)	76	86
**1B**	46 (1267)	25 (754)	0.021	0.094	0.00055	0.0013	6.3 (176)	4.5 (139)	68	82
**2A**	41 (1121)	20 (621)	0.024	0.114	0.00063	0.0016	6.4 (176)	4.5 (139)	64	76
**2B**	43 (1186)	24 (729)	0.023	0.095	0.00059	0.0014	6.4 (176)	4.6 (139)	60	71

a The E-factor and the mass of reaction waste shown do not include the purification waste in the calculation; the number in parentheses is calculated including the purification waste (i.e., aqueous workup and, if necessary, chromatographic separation). Calculations do not include the solvent required to pack the column used for chromatography, as both synthesis methods required this process. Calculated values are approximated to two significant figures.

In general, the peroxidase-catalyzed reactions gave approximately half the E-factor of DAIB-based reactions. One factor contributing to the poorer E-factor of the latter was the use of stoichiometric amounts of DAIB and potassium carbonate (as the base), with every product molecule resulting in the production of two molecules of potassium acetate, CO_2_, and H_2_O as well as one molecule of iodobenzene (Scheme S1). In comparison, the HRP-catalyzed reaction consumed one molecule of H_2_O_2_ and generated two molecules of water. Further, only a very small amount of biocatalyst was necessary (e.g., 2 μg of HRP was used for each preparative scale reaction involving 8 g of aminophenol **1**).

Since the calculation of the E-factor is dependent on what is defined as waste, in this study, anything aside from the reactants, not including water, was categorized as waste. Thus, the main contributor to the superior green metrics is the use of primarily aqueous buffer as the reaction medium in the biocatalytic reaction compared to neat tetrahydrofuran (THF) or dichloromethane (DCM) in the conventional synthesis. Another contributing factor was that the biocatalytic synthesis gives higher isolated yields (the absolute yields were ≥10% higher in all cases, Table 2). This may be due to the higher excess of dienophile used in the biocatalytic reaction that more effectively trapped the highly reactive *o*-quinone imine in the IEDDA reaction. Indeed, it is notable that the green metrics were more favorable in all cases, even when accounting for the larger excess of the dienophile. The production of waste (anything that is not the product or water) can also be quantified by the mass of waste generated (“reaction waste” in Table 2). In all cases, it showed that the biocatalytic reaction produced less waste materials overall per batch of reaction.

Apart from considerations related to the quantities of product and waste, the biocatalytic reaction also offered several further benefits with regard to practical implementation (Table 3). The reactions using DAIB were carried out at 0 °C, with higher temperatures generally resulting in poorer yields,^19^ which has implications in terms of energy efficiency. The reaction medium for the biocatalytic reaction was also less hazardous since a dilute aqueous solution of dioxane would be less toxic compared to DCM, or less flammable compared to THF. Finally, apart from the absolute quantity of waste, the DAIB reactions produced halogenated waste (i.e., iodobenzene, and DCM in those reactions that require it) that would require dedicated disposal measures.

**Table 3:** Summary of Reaction Conditions for the DAIB and HRP/H_2_O_2_ Reactions

oxidant	reaction temperature (°C)	reaction time (h)	solvent	stoichiometric waste
DAIB^19^	0	3	THF or DCM (depending on specific product)	AcOK, PhI, CO_2_, H_2_O
HRP/H_2_O_2_	∼21 (room temperature)	3.5	5% v/v 1,4-dioxane in potassium phosphate buffer	H_2_O

## Conclusions

This report demonstrates the successful development of a biocatalytic oxidation–cycloaddition reaction sequence for the synthesis of 1,4-benzoxazines. This approach was exemplified in a “one-pot, two-step” sequence for the synthesis of fused aromatic morpholinyl compounds bearing selected electron-withdrawing groups in good yields. These yields were superior in comparison to those achieved using the previously reported conventional route with a stoichiometric hypervalent iodine oxidant. It was also found that the regio- and stereochemistry of the final products were unaffected by the use of the enzyme since these aspects were defined by the IEDDA reaction in the second step.

In practical terms, this approach offers the advantages of using readily available aminophenols as the starting material, H_2_O_2_ as a safe and inexpensive oxidant, and HRP as a nontoxic and sustainably sourced catalyst. These benefits are evidenced by direct comparisons of the green metrics between the two synthetic routes (i.e., both use the same starting materials to produce the same products), which show that the biocatalytic reaction is quantifiably a “greener” reaction. Indeed, the E-factor of the biocatalytic reaction is around half and the gRME is around double that of the conventional synthesis. The described route also produces around 30% less waste by mass than the conventional synthesis.

These results suggest that the HRP/H_2_O_2_ combination could be a generalizable replacement to classical stoichiometric oxidants for the generation of other reactive quinone intermediates (e.g., quinone diimines and quinone methides) for a variety of reaction sequences.^4^ Such a capability would not only benefit the synthesis of complex fine chemicals but would also have a range of technological applications such as chemical nanofabrication^39,40^ and bioconjugation.^41^

Further development of this approach would benefit from the development of engineered peroxidase enzymes that would be more stable toward inactivation and redox potentials that are able to oxidize less reactive substrates, thus accessing a wider variety of fused heterocycles in a more sustainable way. In principle, other enzymes capable of oxidizing aminophenols to the corresponding iminoquinones could achieve similar results. For example, oxidase enzymes^42^ that use molecular oxygen as the terminal oxidant could provide one avenue for future investigation.

## Experimental Section

### Materials and Equipment

All solvents and reagents were analytical grade and were purchased from either Merck, Fisher Scientific, or Fluorochem unless stated otherwise. The HRP was purchased from Sigma-Aldrich (catalogue no. P6782) as a lyophilized powder with an enzymatic activity of typically ≥250 U mg^–1^ as measured using a pyrogallol assay. The aminophenols **1**–**3** were produced by hydrogenation of their corresponding nitro analogues (see below).

The HPLC was carried out on an Agilent G1956B LC/MSD SL with a 1200 HPLC System using a Phenomenex Sphereclone C18 4.6 × 250 mm reversed-phase column (solvent A = H_2_O + 0.05% TFA, solvent B = CH_3_CN + 0.05% TFA; 0–13 min B = 5–95%, 13–15 min B = 95%).

### General Procedure for the Preparation of Aminophenols **1**–**3**

The appropriate nitrophenol precursor (1 g) and palladium on activated charcoal (10% Pd by weight, 102 mg) were stirred in methanol (20 mL) under an atmosphere of hydrogen at room temperature for 24 h. The solution was filtered through Celite and dried over anhydrous Na_2_SO_4_, and the solvent was removed under reduced pressure to yield the desired product.

### 3-Amino-4-hydroxybenzonitrile,^43^**1**

4-Hydroxy-3-nitrobenzonitrile (1.00 g, 6.09 mmol) yielded the title compound as a brown solid (805 mg, 6.00 mmol, 98%). ^1^H NMR (400 MHz, CDCl_3_) δ 7.00 (m, 1H), 6.98 (br s, 1H), 6.75 (m, 1H); *m*/*z* (ESI^+^) 135 (MH^+^).

### 2-Amino-4-(trifluoromethyl)phenol,^44^**2**

2-Nitro-4-(trifluoromethyl)phenol (1.00 g, 4.88 mmol) yielded the title compound as an orange solid (836 mg, 4.72 mol, 97%). ^1^H NMR (400 MHz, CDCl_3_) δ 6.98 (s, 1H), 6.93 (d, *J* = 8.0 Hz, 1H), 6.75 (d, *J* = 8.1 Hz, 1H); *m*/*z* (ESI^+^) 178 (MH^+^).

### Ethyl 3-Amino-4-hydroxybenzoate, **3**

Ethyl 4-hydroxy-3-nitrobenzoate (1.00 g, 4.74 mmol) yielded the title compound as a brown solid (817 mg, 4.51 mmol, 95%). ^1^H NMR (400 MHz, CDCl_3_) δ 7.45 (d, *J* = 2.0 Hz, 1H), 7.41 (dd, *J* = 8.2, 2.1 Hz, 1H), 6.74 (d, *J* = 8.2 Hz, 1H), 4.32 (q, *J* = 7.1 Hz, 2H), 1.36 (t, *J* = 7.1 Hz, 3H); *m*/*z* (ESI^+^) 182 (MH^+^).

### General Procedure for Analytical Scale Reactions

The desired dienophile (100 mM), H_2_O_2_ (2.5 mM), and the desired aminophenol (2 mM) were combined in 20 mM potassium phosphate buffer pH 7.4 with 5% v/v 1,4-dioxane to a total reaction volume of 150 μL, 1.5 mL, 30 mL, or 50 mL. Ten microliters of HRP (1 mg mL^–1^ in 20 mM potassium phosphate buffer pH 7.4) was added, and the reaction was shaken at room temperature for 3.5 h. For analysis, a 150 μL sample was removed and quenched by the addition of 75% v*/*v acetonitrile, 25% v*/*v water, 100 mM sodium sulfite, and 100 mM ascorbic acid (300 μL), and the mixture was clarified by centrifugation (10,000 g). The supernatant (10 μL) was then analyzed by HPLC using the mobile phase gradient noted above. Percentage conversions were calculated using the area under the curve (AUC) of the starting material peaks and product peaks along with the AUC of any side product peaks if present. Each reaction was carried out in triplicate, and the results were averaged. For the time-course experiment (Figure S1), individual 150 μL scale reactions were executed, processed, and analyzed by HPLC according to the same procedure at each time interval.

### Preparative Scale Synthesis of 1,4-Benzoxazines

The desired dienophile (100 mM), H_2_O_2_ (25 mM), and the desired aminophenol (20 mM) were combined in 20 mM potassium phosphate buffer pH 7.4 with 5% v/v 1,4-dioxane to a total reaction volume of 50 mL. Two milliliters of HRP (1 μg mL^–1^ in 20 mM potassium phosphate buffer pH 7.4) was then added to start the reaction. Reactions were shaken at 21 °C for 3.5 h. The reaction mixture was extracted with ethyl acetate (25 mL × 3) and the organic extracts were combined and washed with brine (25 mL × 3). The organic phase was then dried over MgSO_4_ and concentrated under reduced pressure. Samples were then analyzed by reversed-phase HPLC, and any reactions that produced the desired product were then subjected to flash column chromatography (*n*-hexane:EtOAc; 2:1) to isolate the product.

### 2,3,3a,9a-Tetrahydro-4*H*-benzo[b]furo[3,2-*e*][1,4]oxazine-6-carbonitrile,^19^**1A**

The title compound was isolated as a brown solid (186 mg, 0.92 mmol, 92%). ^1^H NMR (400 MHz, (CD_3_)_2_CO) δ 7.03–6.92 (m, 2H), 6.86 (d, *J* = 8.2 Hz, 1H), 5.87 (s, 1H), 5.38 (d, *J* = 3.7 Hz, 1H), 4.19 (td, *J* = 8.7, 4.2 Hz, 1H), 4.01 (q, *J* = 8.0 Hz, 1H), 2.21–2.24 (m, 1H), 1.82 (dq, *J* = 12.2, 8.5 Hz, 1H); ^13^C NMR (101 MHz, (CD_3_)_2_CO) δ 145.1, 133.1, 121.6, 119.2, 117.2, 116.7, 104.7, 96.8, 67.5, 53.5, 29.4; *m*/*z* (ESI^+^) 203.0 (MH)^+^.

### 2-Ethoxy-3,4-dihydro-2*H*-benzo[b][1,4]oxazine-6-carbonitrile,^19^**1B**

This compound was isolated as a brown solid (182 mg, 0.89 mmol, 89%). ^1^H NMR (400 MHz, (CD_3_)_2_CO) δ 6.91–6.98 (m, 2H), 6.83–6.89 (m, 1H), 5.34 (s, 1H), 3.64–3.95 (m, 2H), 3.23–3.47 (m, 2H), 1.05–1.29 (m, 3H); ^13^C NMR (400 MHz, (CD_3_)_2_CO) δ 144.9, 135.3, 121.6, 119.2, 117.5, 117.1, 104.6, 95.1, 63.7, 43.7, 14.5; *m*/*z* (ESI^+^) 205.2 (MH)^+^.

### 3,4,4a,10a-Tetrahydro-2*H*,5*H*-benzo[b]pyrano[3,2-*e*][1,4]oxazine-7-carbonitrile, **1C**

The compound was isolated as a dark brown solid (186 mg, 0.86 mmol, 86%). ^1^H NMR (400 MHz, CDCl_3_) δ 6.97 (dd, *J* = 8.3, 1.9 Hz, 1H), 6.82 (d, *J* = 8.3 Hz, 1H), 6.78 (d, *J* = 1.9 Hz, 1H), 5.29 (d, *J* = 2.4 Hz, 1H), 4.00 (ddd, *J* = 11.4, 8.4, 2.9 Hz, 1H), 3.65–3.62 (m, 1H), 3.42 (ddd, *J* = 7.6, 4.2, 2.4 Hz, 1H), 1.75–1.73 (m, 3H), 0.83–0.80 (m, 1H); ^13^C NMR (101 MHz, (CD_3_)_2_CO) δ 170.1, 116.8, 114.4, 94.0, 62.9, 59.7, 54.6, 47.1, 26.0, 21.7, 19.9, 13.6; *m*/*z* (ESI^+^) 217.1 (MH^+^); HRMS (APCI^+^) C_12_H_13_N_2_O_2_^+^ (MH^+^) requires 217.0971, found 217.0972, δ 0.4 ppm.

### 6-(Trifluoromethyl)-2,3,3a,9a-tetrahydro-4*H*-benzo[b]furo[3,2-*e*][1,4]oxazine,^19^**2A**

This compound was isolated as a yellow oil (221 mg, 0.90 mmol, 90%). ^1^H NMR (400 MHz, (CD_3_)_2_CO) δ 7.01–6.95 (m, 2H), 6.88 (d, *J* = 1.3 Hz, 1H), 5.79 (s, 1H), 5.37 (d, *J* = 3.7 Hz, 1H), 4.10–4.30 (m, 2H), 4.01 (q, *J* = 8.0 Hz, 1H), 2.25 (dtd, *J* = 12.2, 7.6, 4.3 Hz, 1H), 1.75–1.95 (m, 1H); ^13^C NMR (101 MHz, (CD_3_)_2_CO) δ 144.1, 132.7, 124.7 (q, *J*_CF_ = 271 Hz), 123.5 (q, *J*_CF_ = 32 Hz), 123.2, 116.6, 114.1, 110.6, 96.7, 67.3, 53.6; ^19^F NMR (376 MHz, (CD_3_)_2_CO) δ −62.11; *m*/*z* (ESI^+^) 246.0 (MH)^+^.

### 2-Ethoxy-6-(trifluoromethyl)-3,4-dihydro-2*H*-benzo[b][1,4]oxazine,^19^**2B**

This compound was isolated as a yellow oil (188 mg, 0.76 mmol, 76%). ^1^H NMR (600 MHz, (CD_3_)_2_CO) δ 6.82 (s, 1H), 6.76–6.77 (m, 2H), 5.34–5.43 (m, 1H), 5.19–5.21 (m, 1H), 3.78 (dq, *J* = 9.7, 7.1 Hz, 1H), 3.60 (dq, *J* = 9.7, 7.1 Hz, 1H), 3.29–3.32 (m, 1H), 3.20–3.23 (m, 1H), 1.06 (t, *J* = 7.1 Hz, 3H); ^13^C NMR (151 MHz, (CD_3_)_2_CO) δ 143.8, 134.8, 124.9 (q, *J*_CF_ = 182 Hz), 123.2 (q, *J*_CF_ = 10 Hz), 116.9, 114.0, 110.0, 94.9, 63.6, 43.5, 14.5; ^19^F NMR (376 MHz, (CD_3_)_2_CO) δ −62.13; *m*/*z* (ESI^+^) 248.0 (MH)^+^.

### 7-(Trifluoromethyl)-3,4,4a,10a-tetrahydro-2*H*,5*H*-benzo[b]pyrano[3,2-*e*][1,4]oxazine, **2C**

This compound was isolated as a yellow oil (218 mg, 0.84 mmol, 84%). ^1^H NMR (400 MHz, (CD_3_)_2_CO) δ 7.27–7.45 (m, 1H), 7.01–7.27 (m, 1H), 6.95–7.00 (m, 1H), 5.32 (d, *J* = 2.2 Hz, 1H), 4.07 (q, *J* = 7.1 Hz, 1H), 3.85–4.00 (m, 2H), 3.61–3.75 (m, 1H), 3.44–3.60 (m, 1H), 2.10 (q, *J* = 7.1 Hz, 1H), 1.21 (t, *J* = 7.1 Hz, 1H); ^13^C NMR (400 MHz, (CD_3_)_2_CO) δ 144.14, 132.96, 123.6 (q, *J*_CF_ = 315 Hz), 119.89, 119.5 (q, *J*_CF_ = 40 Hz), 116.90, 113.12, 94.62, 63.51, 47.90, 26.77, 22.46; ^19^F NMR (376 MHz, (CD_3_)_2_CO) δ −61.55; *m*/*z* (ESI^+^) 260 (MH^+^); HRMS (APCI^+^) C_12_H_13_F_3_NO_4_^+^ (MH^+^) requires 260.0892, found 260.0893, δ 0.4 ppm.

### Ethyl 2,3,3a,9a-Tetrahydro-4*H*-benzo[b]furo[3,2-*e*][1,4]oxazine-6-carboxylate, **3A**

This compound was isolated as a yellow solid (217 mg, 0.87 mmol, 87%). ^1^H NMR (400 MHz, (CD_3_)_2_CO) δ 7.36 (d, *J* = 2.1 Hz, 1H), 7.30 (dd, *J* = 8.3, 2.1 Hz, 1H), 6.80 (d, *J* = 8.3 Hz, 1H), 5.39 (d, *J* = 3.8 Hz, 1H), 4.28 (q, *J* = 7.1 Hz, 1H), 4.22–4.05 (m, 2H), 4.00 (ap q, *J* = 8.0 Hz, 2H), 2.10–2.25 (m, 1H), 1.80–1.90 (m, 1H), 1.33 (t, *J* = 7.1 Hz, 3H); ^13^C NMR (101 MHz, (CD_3_)_2_CO) δ 165.9, 145.6, 131.8, 124.0, 119.5, 116.1, 115.3, 97.1, 67.4, 60.0, 53.7, 28.4, 13.8; *m*/*z* (ESI^+^) 250 (MH^+^); HRMS (APCI^+^) C_13_H_16_NO_4_^+^ (MH^+^) requires 250.1073, found 250.1074, δ 0.4 ppm.

### Ethyl 2-Ethoxy-3,4-dihydro-2*H*-benzo[b][1,4]oxazine-6-carboxylate, **3B**

This compound was isolated as a yellow solid (168 mg, 0.67 mmol, 67%). ^1^H NMR (400 MHz, CDCl_3_) δ 7.33–7.37 (m, 2H), 6.76 (dd, *J* = 8.3, 3.0 Hz, 1H), 5.19 (d, *J* = 3.6 Hz, 1H), 4.21–4.27 (m, 2H), 3.87 (m, 2H), 3.63 (dq, *J* = 9.7, 7.1 Hz, 1H), 3.32–3.24 (m, 2H), 1.30–1.27 (m, 3H), 1.17–1.13 (m, 3H); ^13^C NMR (101 MHz, (CD_3_)_2_CO) δ 162.4, 125.5, 119.5, 118.2, 116.4, 115.9, 95.3, 63.6, 60.7, 44.0, 14.6, 13.8, 13.7; *m*/*z* (ESI^+^) 252 (MH^+^); HRMS (APCI^+^) C_13_H_18_NO_4_^+^ (MH^+^) requires 252.1230, found 252.1230; δ < 0.1 ppm.

### Ethyl 3,4,4a,10a-Tetrahydro-2*H*,5*H*-benzo[b]pyrano[3,2-*e*][1,4]oxazine-7-carboxylate, **3C**

This compound was isolated as an ochre solid (111 mg, 0.42 mmol, 42%). ^1^H NMR (400 MHz, (CD_3_)_2_CO) δ 8.46 (d, *J* = 2.2 Hz, 1H), 7.94 (dd, *J* = 8.7, 2.2 Hz, 1H), 7.07 (d, *J* = 8.7 Hz, 1H), 4.25 (q, *J* = 7.1 Hz, 2H), 1.26 (t, *J* = 7.1 Hz, 3H); ^13^C NMR (151 MHz, (CD_3_)_2_CO) δ 165.1, 145.8, 131.0, 129.2, 122.4, 118.7, 117.8, 116.7, 63.2, 60.6, 59.6, 19.9, 13.7, 13.2; *m*/*z* (ESI^+^) 264 (MH^+^); HRMS (APCI^+^) C_14_H_18_NO_4_^+^ (MH^+^) requires 264.1230, found 264.1230, δ < 0.1 ppm.

### 6-Chloro-2,3,3a,9a-tetrahydro-4*H*-benzo[b]furo[3,2-*e*][1,4]oxazine, **4A**

This compound was isolated as a brown solid (108 mg, 0.51 mmol, 51%). ^1^H NMR (400 MHz, (CD_3_)_2_CO) δ 6.71 (d, *J* = 8.5 Hz, 1H), 6.68 (d, *J* = 2.4 Hz, 1H), 6.54 (dd, *J* = 8.4, 2.4 Hz, 1H), 5.64 (s, 1H), 5.30 (d, *J* = 3.8 Hz, 1H), 4.15 (td, *J* = 8.6, 4.5 Hz, 1H), 4.07 (td, *J* = 7.9, 3.7 Hz, 1H), 3.97 (q, *J* = 7.8 Hz, 1H), 2.23 (dtd, *J* = 12.2, 7.6, 4.5 Hz, 1H), 1.83 (dq, *J* = 12.1, 8.2 Hz, 1H); ^13^C NMR (151 MHz, (CD_3_)_2_CO) δ 149.8, 129.3, 128.3, 127.2, 117.4, 103.8, 67.5, 59.7, 19.9, 13.6; *m*/*z* (ESI^+^) 212 (MH^+^); HRMS (APCI^+^) C_10_H_11_ClNO_2_^+^ (MH^+^) requires 212.0472, found 212.0473, δ 0.5 ppm.

## Supporting Information Available

Supporting results (Figures S1 and S2 and Table S1) and scheme (Scheme S1), ^1^H NMR assignments, and compound characterization data (Figures S3–S40) (PDF)

## Footnotes

a Throughout this article, the 1,4-benzoxazine products are identified by two-character designations corresponding to the combinations of the aminophenol and dienophile used in the reaction.
