# Supplementary material for: Biocatalytic Generation of o-Quinone Imines in the Synthesis of 1,4-Benzoxazines and Its Comparative Green Chemistry Metrics
Source: ACS Sustain Chem Eng. 2024 Feb 2;12(7):2678–85. doi: 10.1021/acssuschemeng.3c06758 (PMC10880089; doi:10.1021/acssuschemeng.3c06758)
Supplement: Supplementary file 1 — sc3c06758_si_001.pdf [file sc3c06758_si_001.pdf]

Supporting Information

Biocatalytic Generation of *o*-Quinone Imines in the Synthesis of 1,4-Benzoxazines and Its Comparative Green Chemistry Metrics

Maryam Tehami[a,b,†], Hasan Tanvir Imam[a,b,†], Iskandar Abdullah[a,b,c,†], Joseph Hosford[a,b,†], Xiao Juie Wong[b], Noorsaadah Abdul Rahman[d] and Lu Shin Wong[a,b,*]

[a] Manchester Institute of Biotechnology, University of Manchester, 131 Princess Street, M1 7DN, Manchester, United Kingdom

[b] Department of Chemistry, University of Manchester, Oxford Road, Manchester, M13 9PL, United Kingdom

[c] Department of Chemistry, Faculty of Science, Universiti Malaya, 50603 Kuala Lumpur, Malaysia

[d] Institute for Advanced Studies, Universiti Malaya, 50603 Kuala Lumpur, Malaysia

* Corresponding author:

Lu Shin Wong, tel.: +44 161 529 4357; e-mail: l.s.wong@manchester.ac.uk

† These authors contributed equally to the work

Table of Contents: **Page**

Supplementary Results and Discussion (Figures S1, S2; Table S1, Scheme S1)	S2
1H NMR Assignments	S6
Compound Characterization Data (Figures S3 – S41)	S10

Supporting Information contains 30 pages including 41 Figures, 1 Table and 1 Scheme.

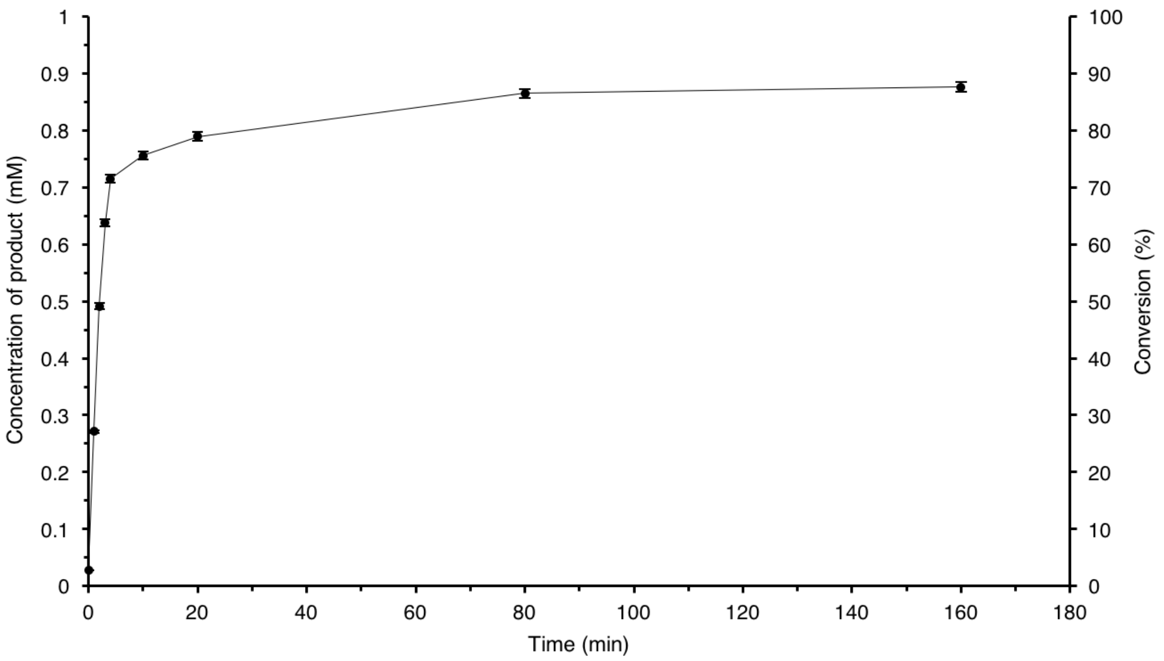

Figure S1. Plot of product conversion against time for the formation of **1A**.

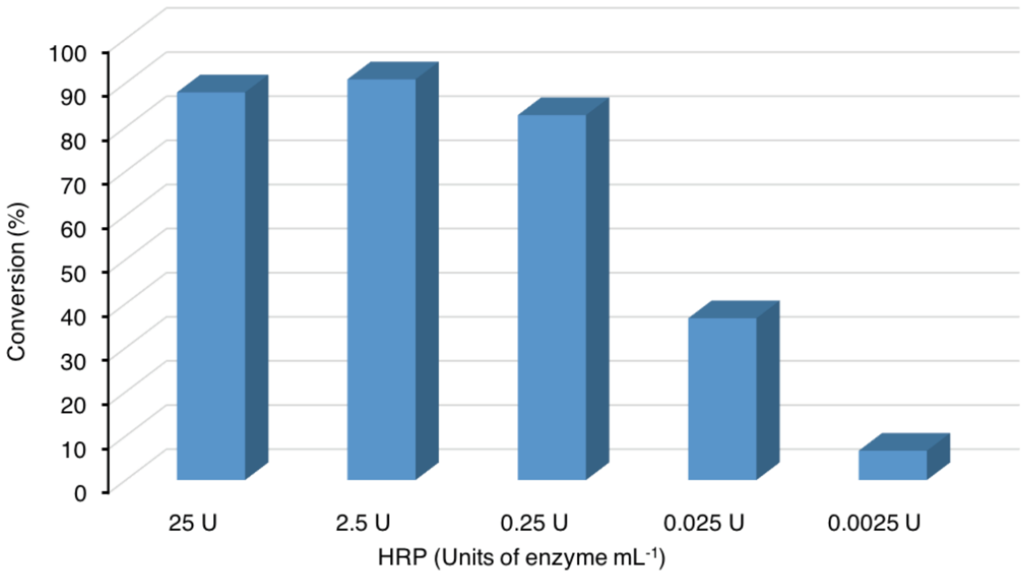

Figure S2. Bar chart of product **1A** conversion against concentration of HRP.

Table S1. Percentage conversions and isolated yields of the oxidation-IEDDA reaction sequence for each pair of aminophenol and dienophile. The conversions are shown with the calculated standard deviation from experimental triplicates. ND = Not determined. Color key: green ≥ 75 %; yellow ≥ 50 % to < 75 %; pink < 50 %; grey = no product detected or isolated.

Aminophenol	Dienophile	Anticipated product	Conversion (%) at various reaction scales				Isolated Yield (%)
			0.3 nmol / 150 µL	3.0 nmol / 1.5 mL	60 µmol / 30 mL	1 mmol / 50 mL	
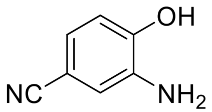 **1**	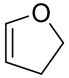 **A**	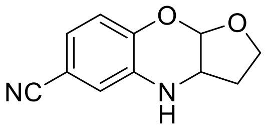 **1A**	100 ± 0.0	96 ± 1.4	99 ± 0.6	100 ± 0.0	92
	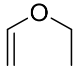 **B**	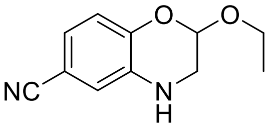 **1B**	52 ± 5.3	90 ± 0.8	91 ±1.6	96 ± 3.5	89
	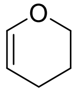 **C**	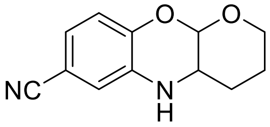 **1C**	100 ± 0.0	100 ± 0.0	69 ± 3.0	94 ± 3.5	86
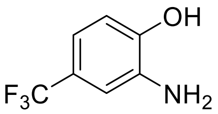 **2**	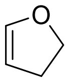 **A**	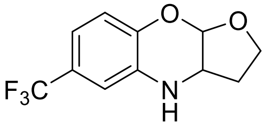 **2A**	95 ± 2.9	75 ± 2.9	99 ± 1.0	99 ± 1.6	90
	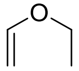 **B**	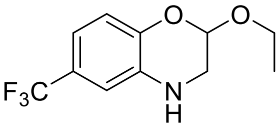 **2B**	68 ± 6.3	79 ± 18.7	82 ± 2.1	90 ± 8.9	76

	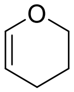 **C**	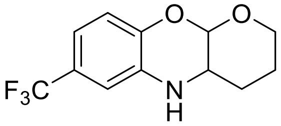 **2C**	96 ± 1.8	83 ± 6.5	70 ± 5.9	90 ± 8.9	84
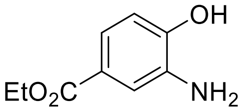 **3**	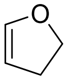 **A**	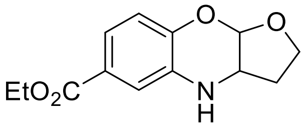 **3A**	34 ± 4.7	70 ± 5.9	98 ± 0.8	92 ± 2.5	87
	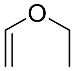 **B**	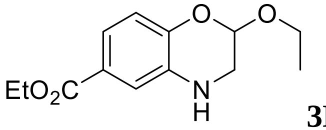 **3B**	14 ± 8.8	37 ± 4.4	72 ± 11.5	83 ± 4.6	67
	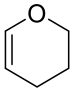 **C**	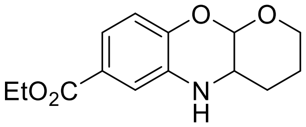 **3C**	61 ± 22.0	0	63 ± 4.4	64 ± 5.4	42
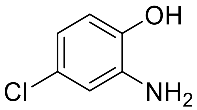 **4**	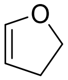 **A**	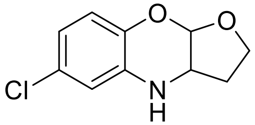 **4A**	96 ± 2.6	95 ± 0.5	67 ± 8.1	56 ± 7.6	51
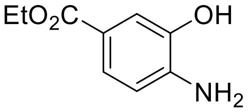 **5**	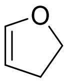 **A**	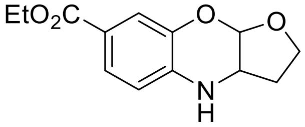 **5A**	43 ± 1.2	63 ±7.3	29 ± 5.8	0	ND
	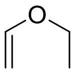 **B**	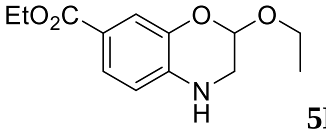 **5B**	0	0	14 ± 4.5	0	ND
	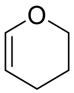 **C**	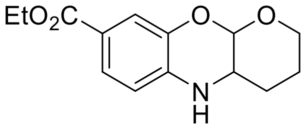 **5C**	0	0	0	5 ± 6.7	ND

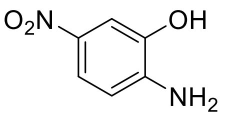 6	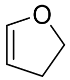 A	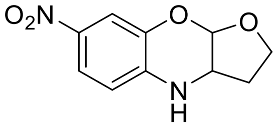 6A	10 ± 3.1	51 ± 7.4	43 ± 5.0	6 ± 5.7	0
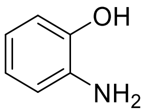 7	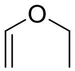 B	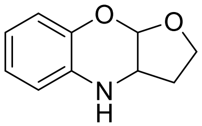 7A	0	ND	ND	ND	ND
	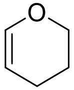 C	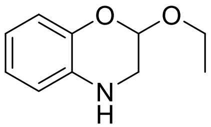 7B	0	ND	ND	ND	ND
	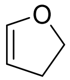 A	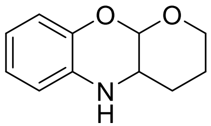 7C	0	ND	ND	ND	ND

(A)

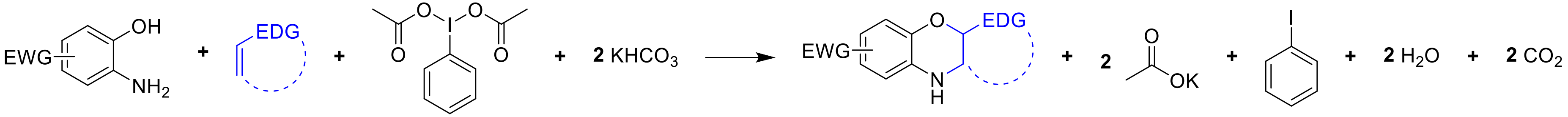

(B)

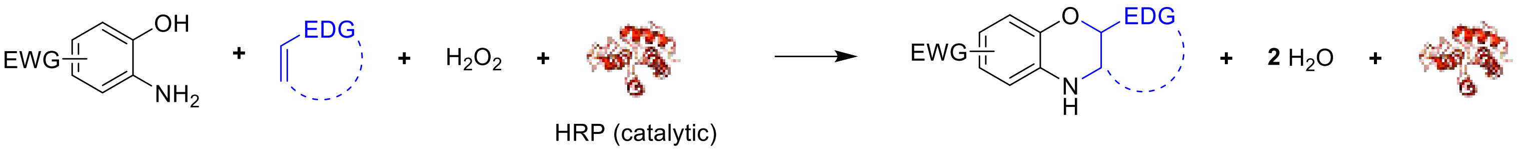

Scheme S1. Schemes showing the overall stoichiometry of the "one-pot" reaction starting materials, products and by-products where: (A) DAIB was used as the oxidant, or (B) HRP/H_2O_2 was used.

^{1}H NMR Assignments

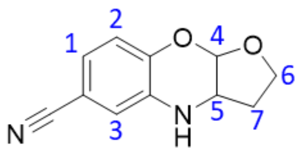

2,3,3a,9a-Tetrahydro-4H-benzo[b]furo[3,2-e][1,4]oxazine-6-carbonitrile, 1A. ^{1}H NMR (400 MHz, $(CD_3)_2CO$) δ 7.03 – 6.92 (m, 2H, 1 and 3), 6.86 (d, *J* = 8.2 Hz, 1H, 2), 5.87 (s, 1H, 4), 5.38 (d, *J* = 3.7 Hz, 1H, 5), 4.19 (td, *J* = 8.7, 4.2 Hz, 1H, 6a), 4.01 (q, *J* = 8.0 Hz, 1H, 6b), 2.21–2.24 (m, 1H, 7a), 1.82 (dq, *J* = 12.2, 8.5 Hz, 1H, 7b).

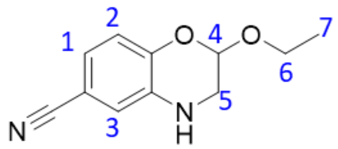

2-Ethoxy-3,4-dihydro-2H-benzo[b][1,4]oxazine-6-carbonitrile, 1B. ^{1}H NMR (400 MHz, $(CD_3)_2CO$) δ 6.91 - 6.98 (m, 2H, 1 and 3), 6.83 - 6.89 (m, 1H, 2), 5.34 (s, 1H, 4), 3.64 - 3.95 (m, 2H, 6), 3.23 - 3.47 (m, 2H, 5), 1.05 - 1.29 (m, 3H, 7).

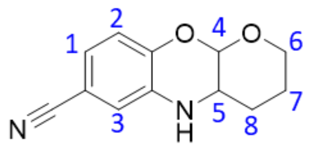

3,4,4a,10a-Tetrahydro-2H,5H-benzo[b]pyrano[3,2-e][1,4]oxazine-7-carbonitrile, 1C. ^{1}H NMR (400 MHz, $CDCl_3$) δ 6.97 (dd, *J* = 8.3, 1.9 Hz, 1H, 3), 6.82 (d, *J* = 8.3 Hz, 1H, 1), 6.78 (d, *J* = 1.9 Hz, 1H, 2), 5.29 (d, *J* = 2.4 Hz, 1H, 4), 4.00 (m, 1H, 5), 3.65 – 3.62 (m, 1H, 6a), 3.42 (ddd, *J* = 7.6, 4.2, 2.4 Hz, 1H, 6b), 1.75 – 1.73 (m, 3H, 7 and 8a), 0.83 – 0.80 (m, 1H, 8b).

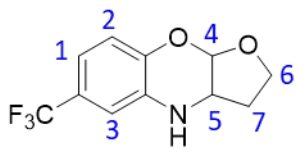

6-(Trifluoromethyl)-2,3,3a,9a-tetrahydro-4H-benzo[b]furo[3,2-e][1,4]oxazine, 2A. ^{1}H NMR (400 MHz, $(CD_3)_2CO$) δ 7.01 – 6.95 (m, 2H, 2 and 3), 6.88 (d, *J* = 1.3 Hz, 1H, 1), 5.79 (s, 1H, NH), 5.37 (d, *J* = 3.7 Hz, 1H, 4), 4.10 – 4.30 (m, 2H, 6), 4.01 (q, *J* = 8.0 Hz, 1H, 5), 2.25 (dtd, *J* = 12.2, 7.6, 4.3 Hz, 1H, 7a), 1.75 – 1.95 (m, 1H, 7b).

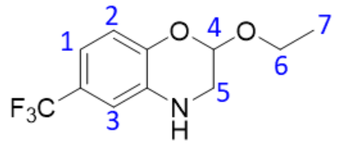

2-Ethoxy-6-(trifluoromethyl)-3,4-dihydro-2H-benzo[b][1,4]oxazine, 2B. ^{1}H NMR (600 MHz, $(CD_3)_2CO$) δ 6.82 (s, 1H, 3), 6.76 – 6.77 (m, 2H, 1 and 2), 5.34 – 5.43 (m, 1H, NH), 5.19 – 5.21 (m, 1H, 4), 3.78 (dq, *J* = 9.7, 7.1 Hz, 1H, 6a), 3.60 (dq, *J* = 9.7, 7.1 Hz, 1H, 6b), 3.29 - 3.32 (m, 1H, 5a), 3.20 - 3.23 (m, 1H, 5b), 1.06 (t, *J* = 7.1 Hz, 3H, 7).

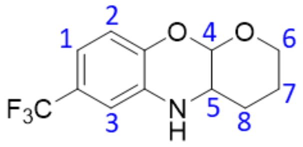

7-(Trifluoromethyl)-3,4,4a,10a-tetrahydro-2H,5H-benzo[b]pyrano[3,2-e][1,4]oxazine, 2C. ^{1}H NMR (400 MHz, $(CD_3)_2CO$) δ 7.27 – 7.45 (m, 1H, 3), 7.01 - 7.27 (m, 1H, 2), 6.95 – 7.00 (m, 1H, 1), 5.32 (d, *J* = 2.2 Hz, 1H, 4), 4.07 (q, *J* = 7.1 Hz, 1H, 5), 3.85 - 4.00 (m, 2H, 6), 3.61 – 3.75 (m, 1H, 7a), 3.44 – 3.60 (m, 1H, 7b), 2.10 (q, *J* = 7.1 Hz, 1H, 8a), 1.21 (t, *J* = 7.1 Hz, 1H, 8b).

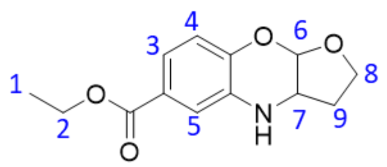

Ethyl 2,3,3a,9a-tetrahydro-4H-benzo[b]furo[3,2-e][1,4]oxazine-6-carboxylate, 3A. ^{1}H NMR (400 MHz, $(CD_3)_2CO$) δ 7.36 (d, *J* = 2.1 Hz, 1H, 5), 7.30 (dd, *J* = 8.3, 2.1 Hz, 1H, 3), 6.80 (d, *J* = 8.3 Hz, 1H, 4), 5.39 (d, *J* = 3.8 Hz, 1H, 6), 4.28 (q, *J* = 7.1 Hz, 1H, 7), 4.22 – 4.05 (m, 2H, 8), 4.00 (ap q, *J* = 8.0 Hz, 2H, 9), 2.10 – 2.25 (m, 1H, 2a), 1.80 – 1.90 (m, 1H, 2b), 1.33 (t, *J* = 7.1 Hz, 3H, 1).

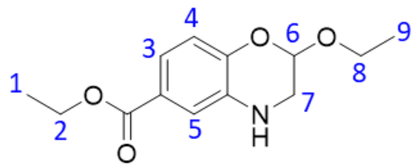

Ethyl 2-ethoxy-3,4-dihydro-2H-benzo[b][1,4]oxazine-6-carboxylate, 3B. ^{1}H NMR (400 MHz, $CDCl_3$) δ 7.33 – 7.37 (m, 2H, 3 and 5), 6.76 (dd, *J* = 8.3, 3.0 Hz, 1H, 4), 5.19 (d, *J* = 3.6 Hz, 1H, 6), 4.21 – 4.27 (m, 2H, 2), 3.87 (m, 2H, 7a and NH), 3.63 (dq, *J* = 9.7, 7.1 Hz, 1H, 7b), 3.32 – 3.24 (m, 2H, 8), 1.30 – 1.27 (m, 3H, 1), 1.17 – 1.13 (m, 3H, 9).

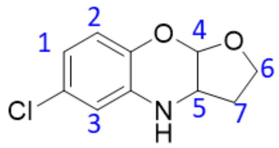

6-chloro-2,3,3a,9a-tetrahydro-4H-benzo[b]furo[3,2-e][1,4]oxazine, 4A. ^{1}H NMR (400 MHz, $(CD_3)_2CO$) δ 6.71 (d, *J* = 8.5 Hz, 1H, 3), 6.68 (d, *J* = 2.4 Hz, 1H, 1), 6.54 (dd, *J* = 8.4, 2.4 Hz, 1H, 2), 5.64 (s, 1H, 4), 5.30 (d, *J* = 3.8 Hz, 1H, NH), 4.15 (dt, *J* = 8.3, 4.1 Hz, 1H, 6a),

4.07 (dt, J = 8.3, 4.1 Hz, 1H, 6b), 3.97 (m, 1H, 5), 2.15 – 2.30 (m, 1H, 7a), 1.75 – 1.90 (m, 1H, 7b).

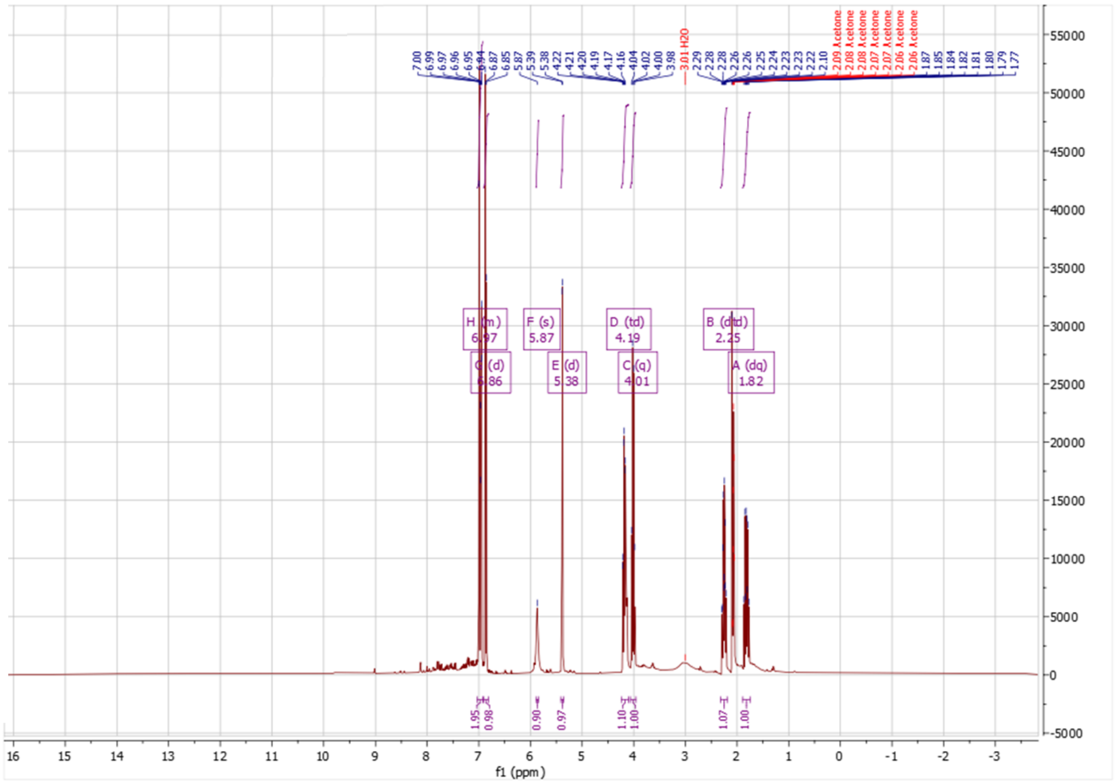

Figure S3. ^{1}H NMR Spectrum of **1A** in hexadeuteroacetone.

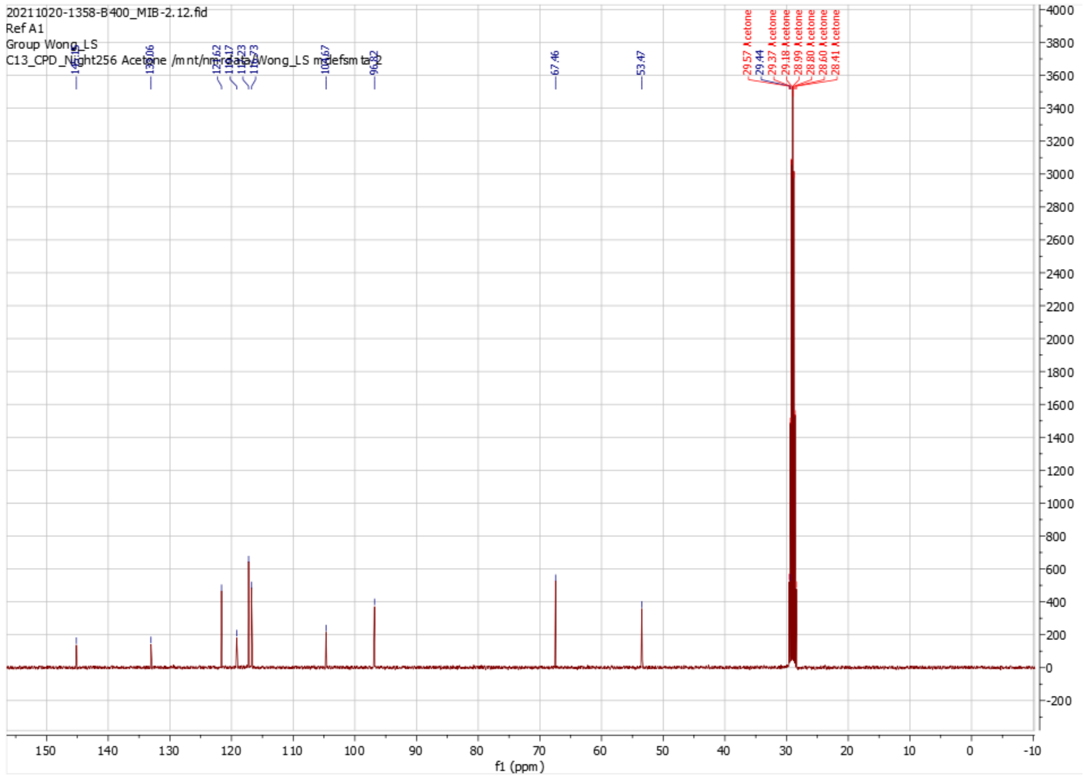

Figure S4. ^{13}C NMR Spectrum of **1A** in hexadeuteroacetone.

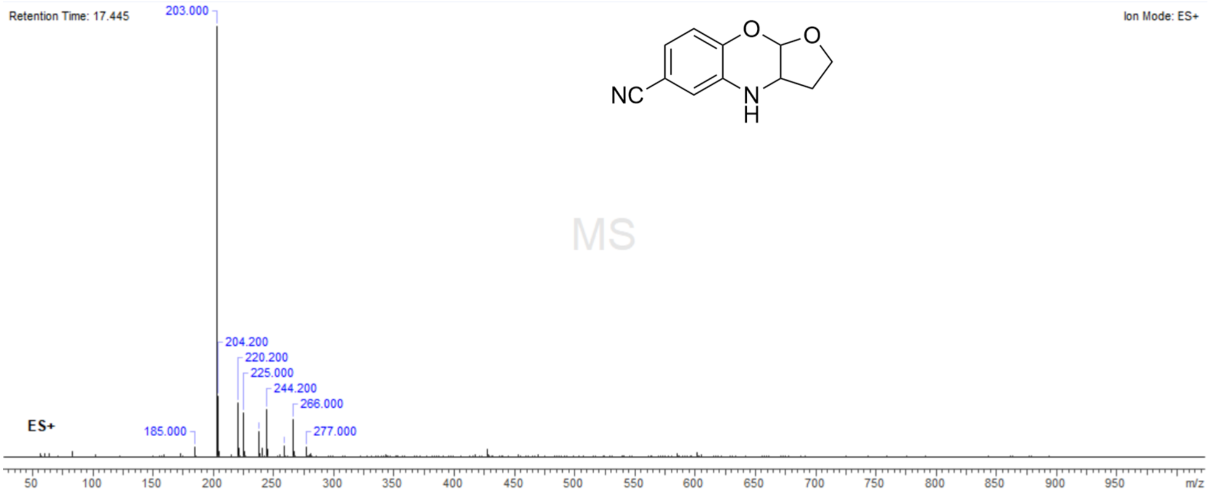

Figure S5. Positive mode electrospray mass spectrum of **1A.**

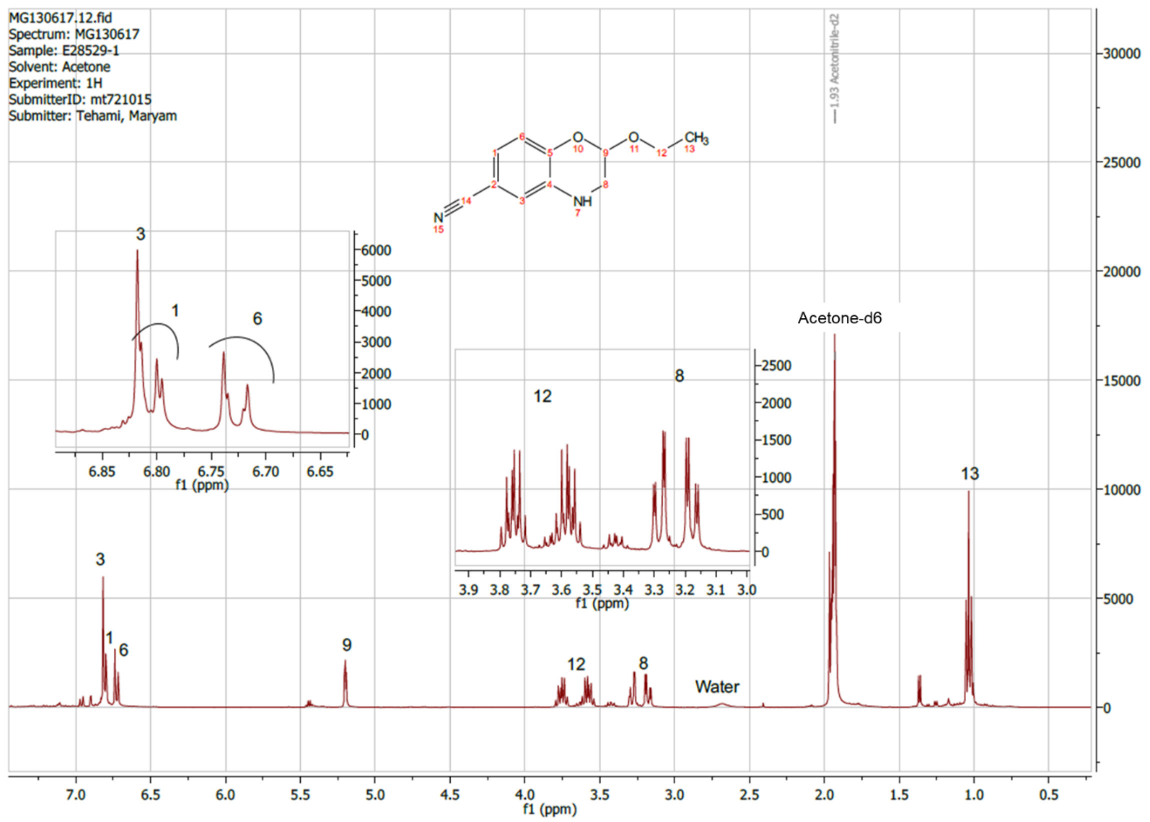

Figure S6. ^{1}H NMR Spectrum of **1B** in hexadeuteroacetone.

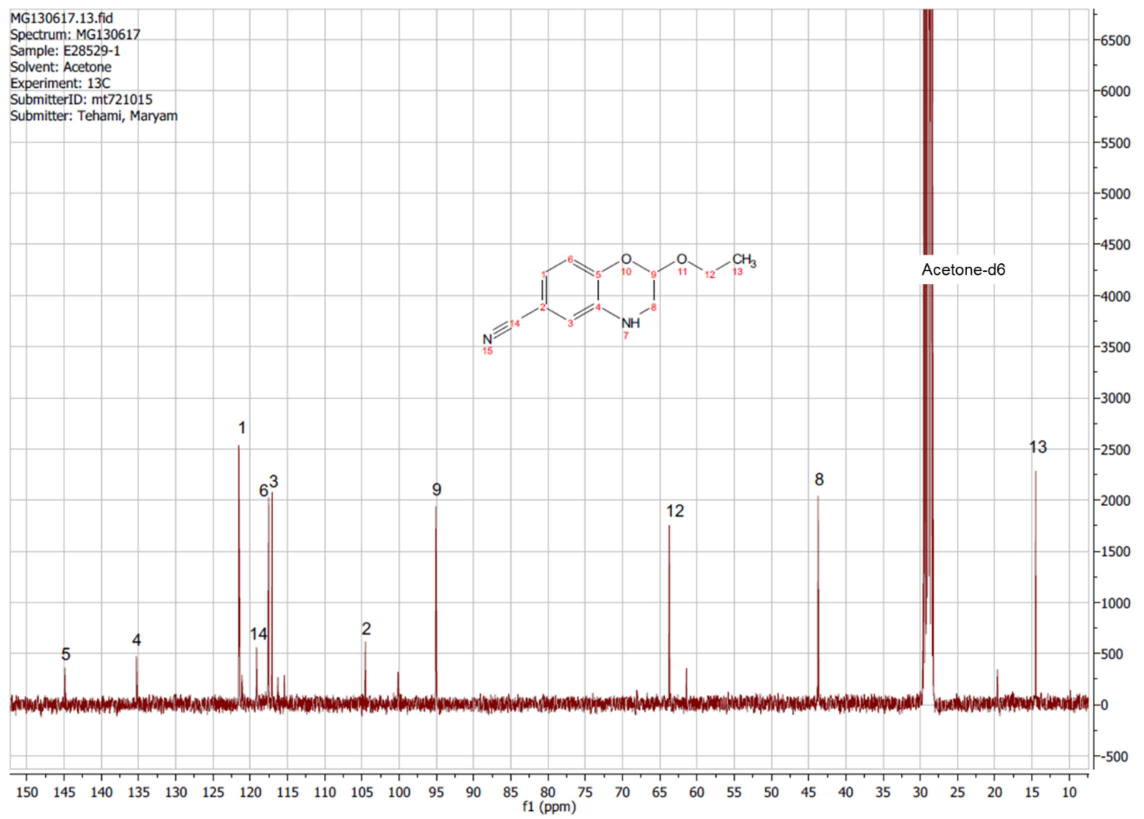

Figure S7. ^{13}C NMR Spectrum of **1B** in hexadeuteroacetone.

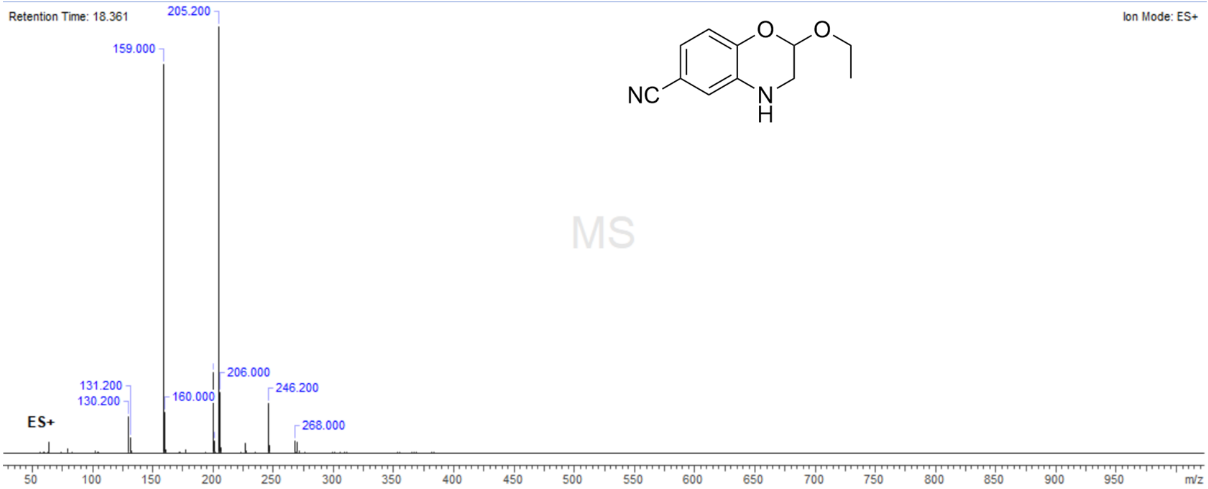

Figure S8. Positive mode electrospray mass spectrum of **1B.**

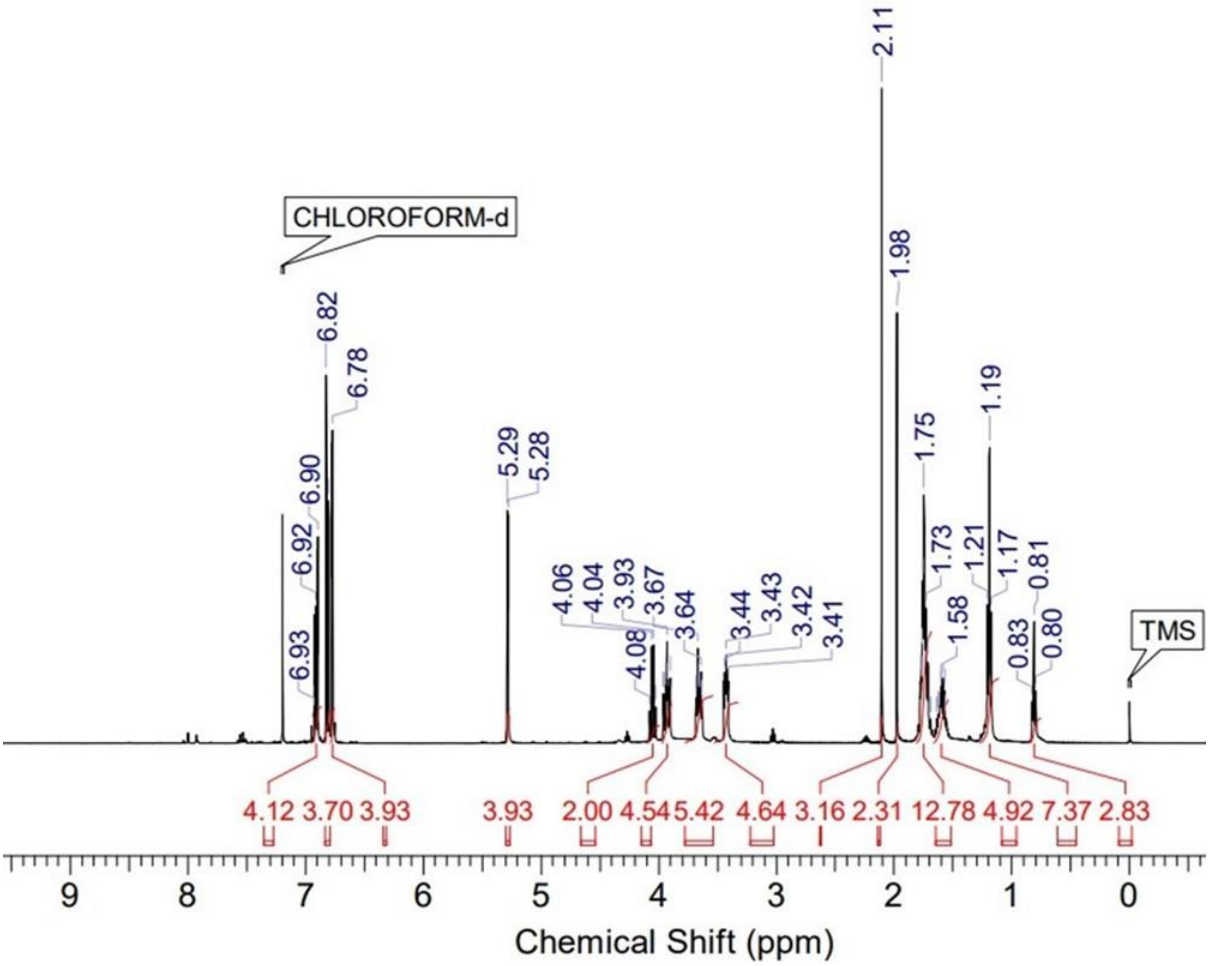

Figure S9. ^{1}H NMR Spectrum of **1C** in deuterochloroform.

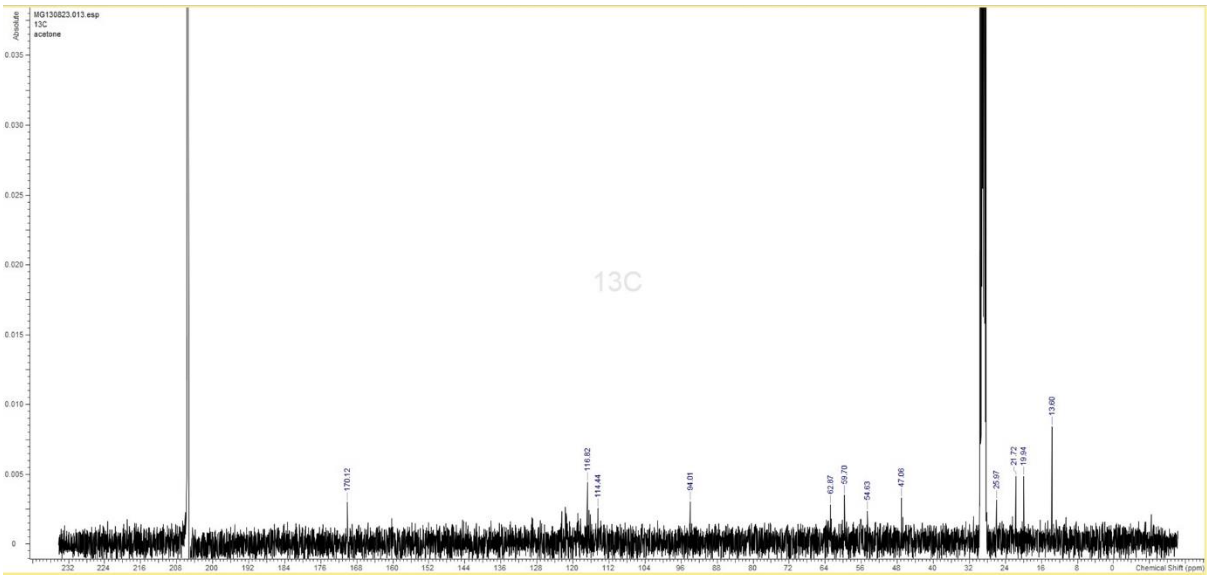

Figure S10. ^{13}C NMR Spectrum of **1C** in hexadeuteroacetone.

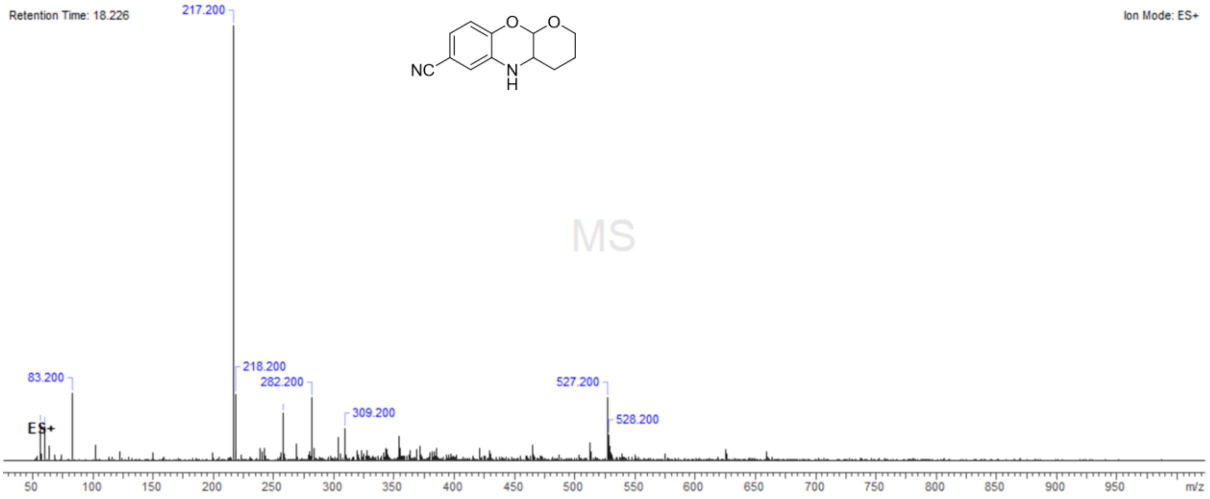

Figure S11. Positive mode electrospray mass spectrum of **1C.**

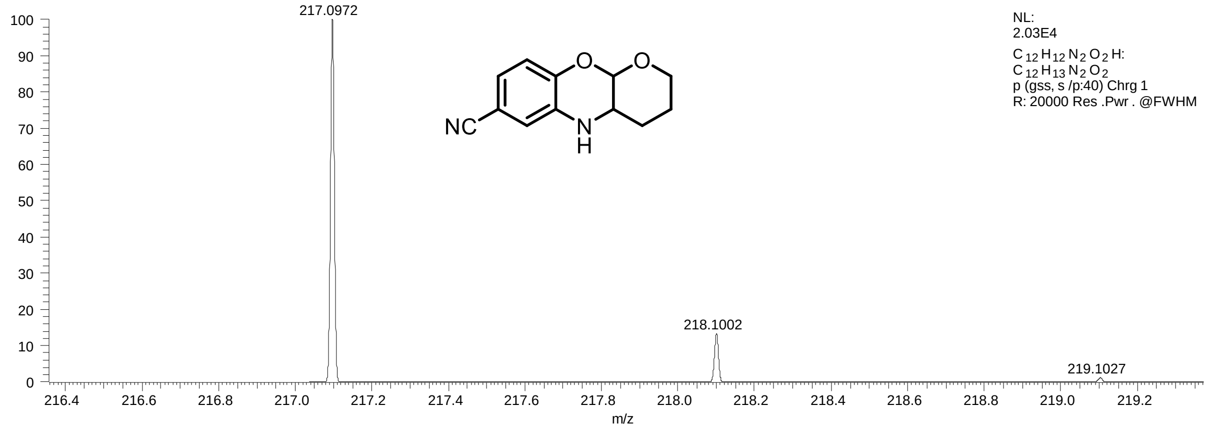

Figure S12. High Resolution Mass spectrum of **1C.**

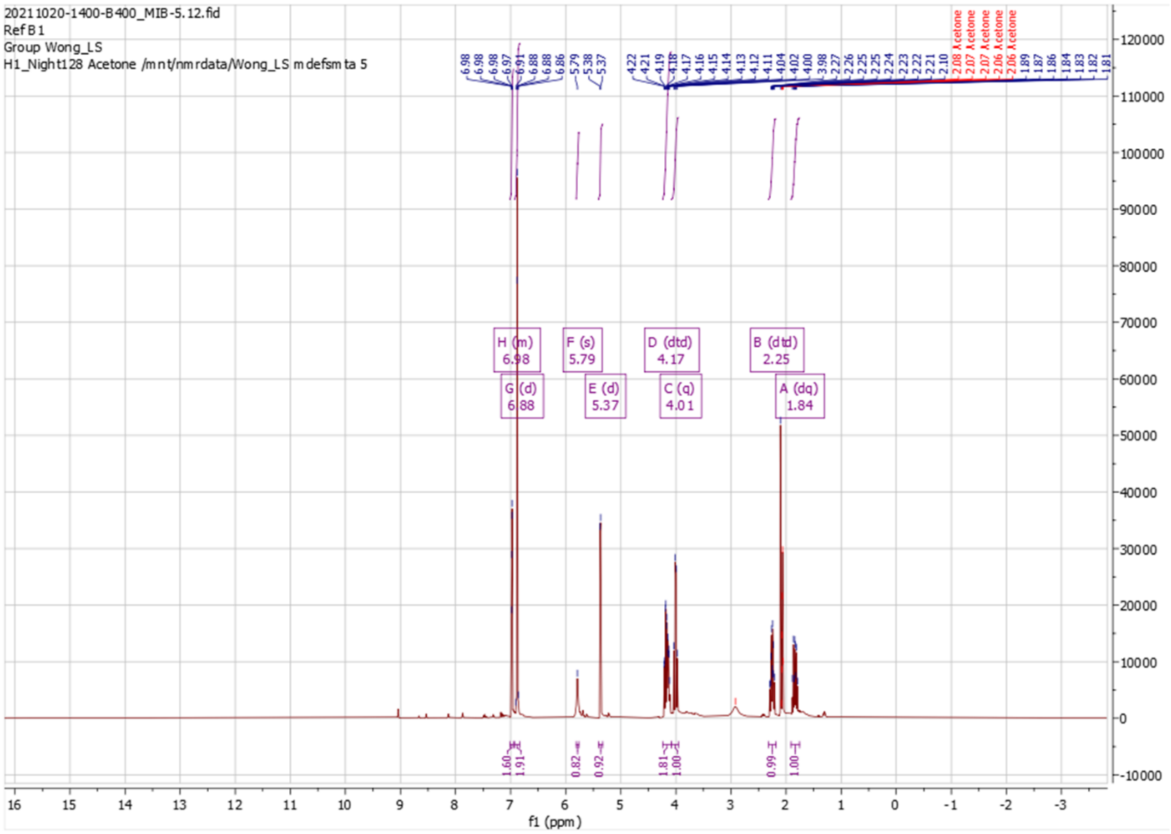

Figure S13. ^{1}H NMR Spectrum of **2A** in hexadeuteroacetone.

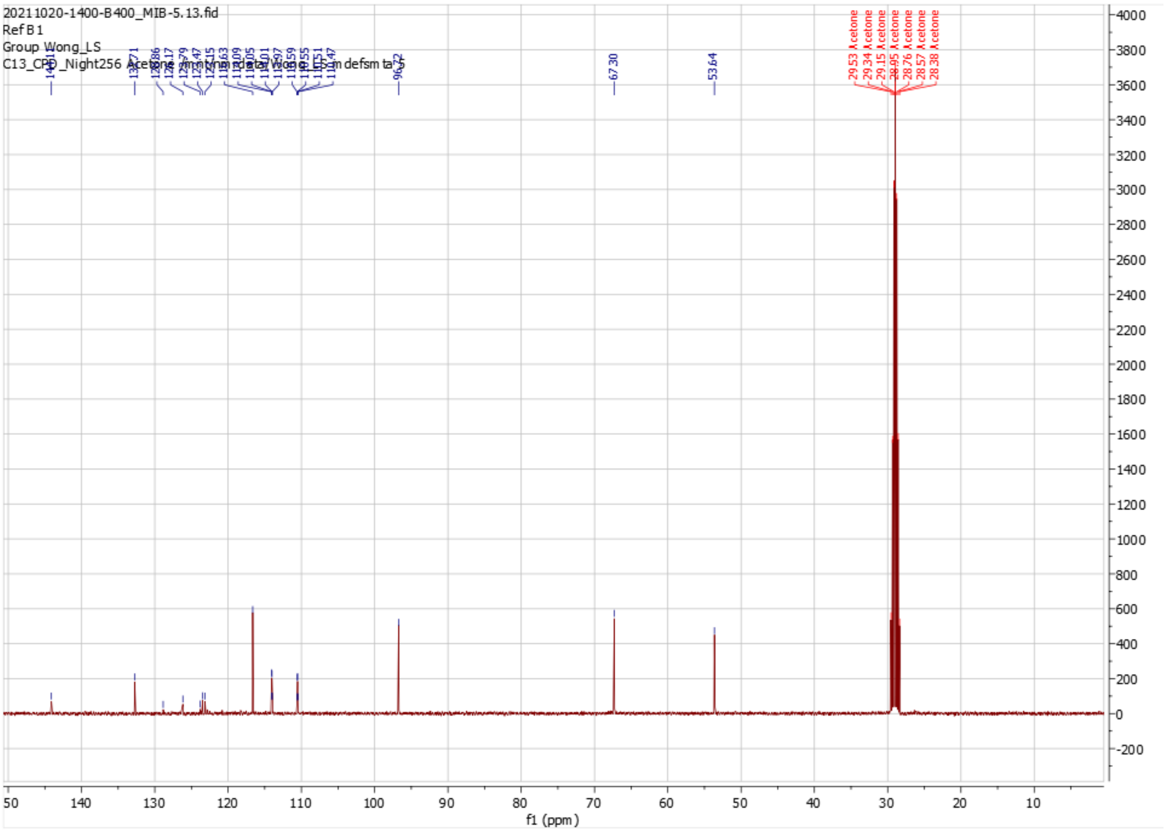

Figure S14. ^{13}C NMR Spectrum of **2A** in hexadeuteroacetone.

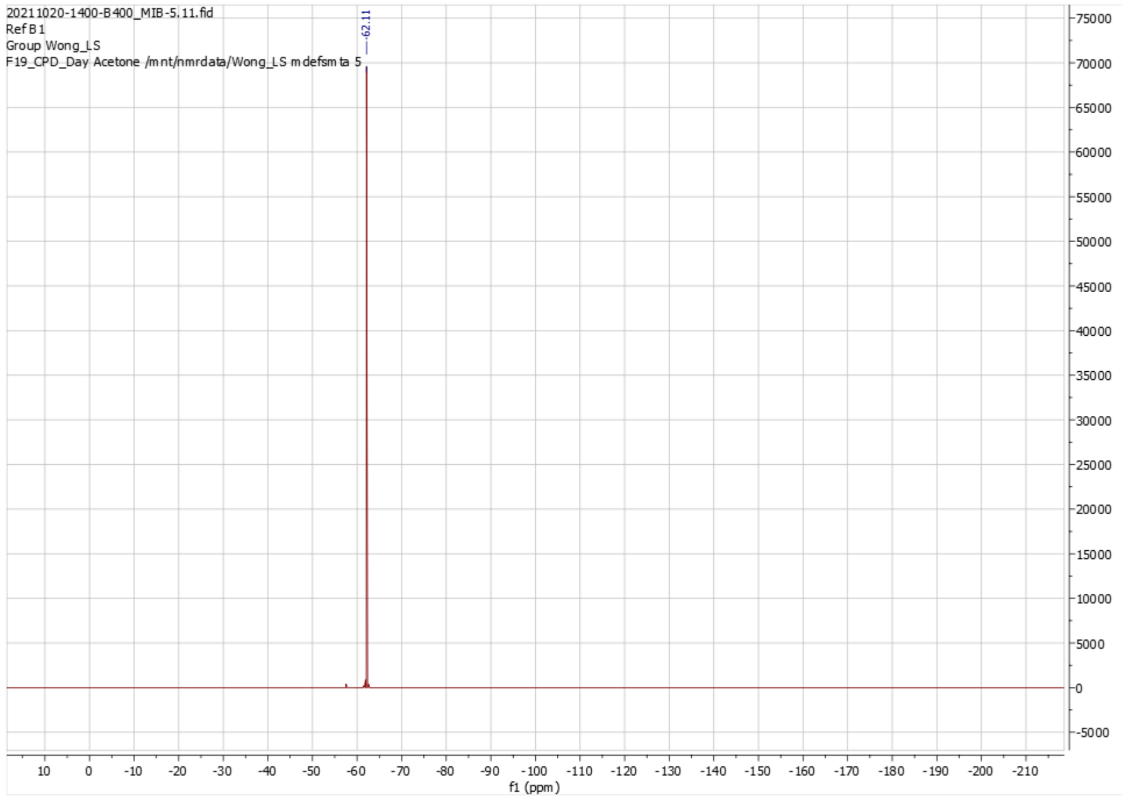

Figure S15. ^{19}F NMR Spectrum of **2A** in hexadeuteroacetone.

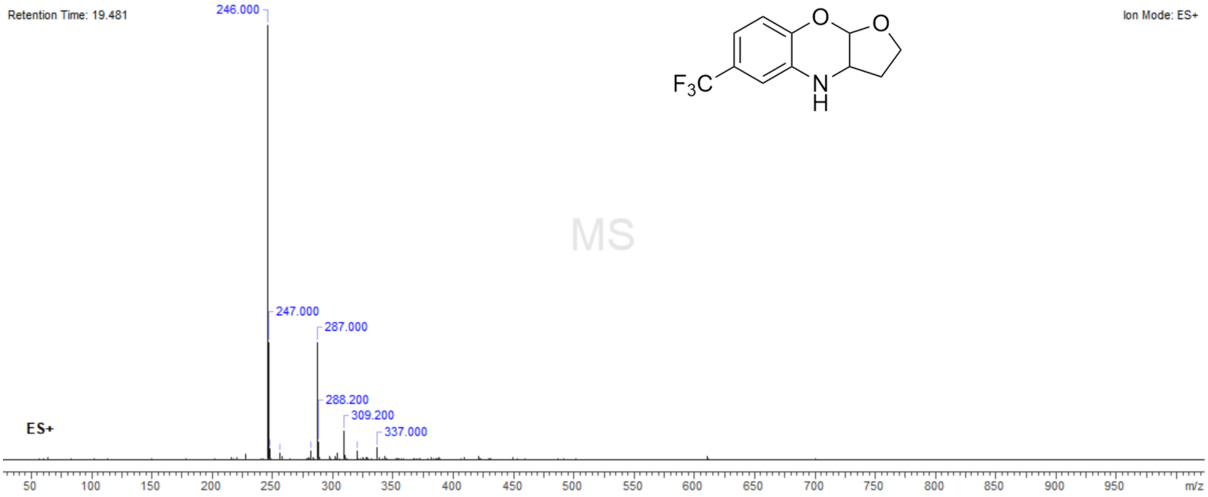

Figure S16. Positive mode electrospray mass spectrum of **2A.**

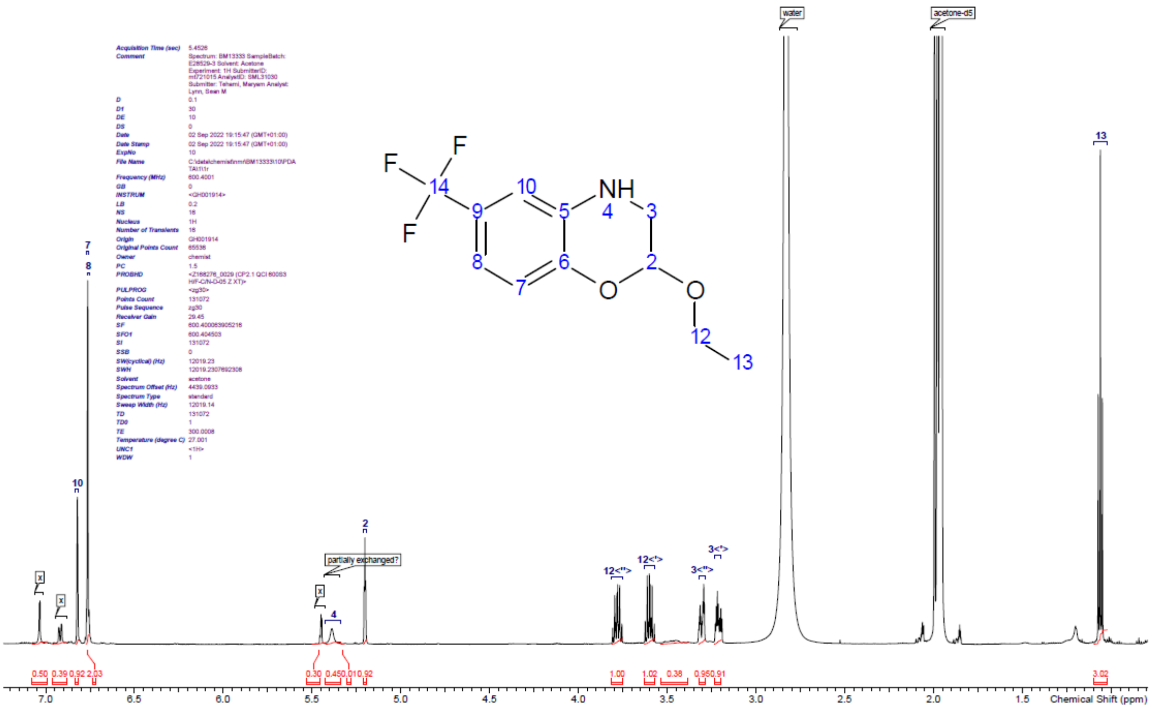

Figure S17. ^{1}H NMR Spectrum of **2B** in hexadeuteroacetone.

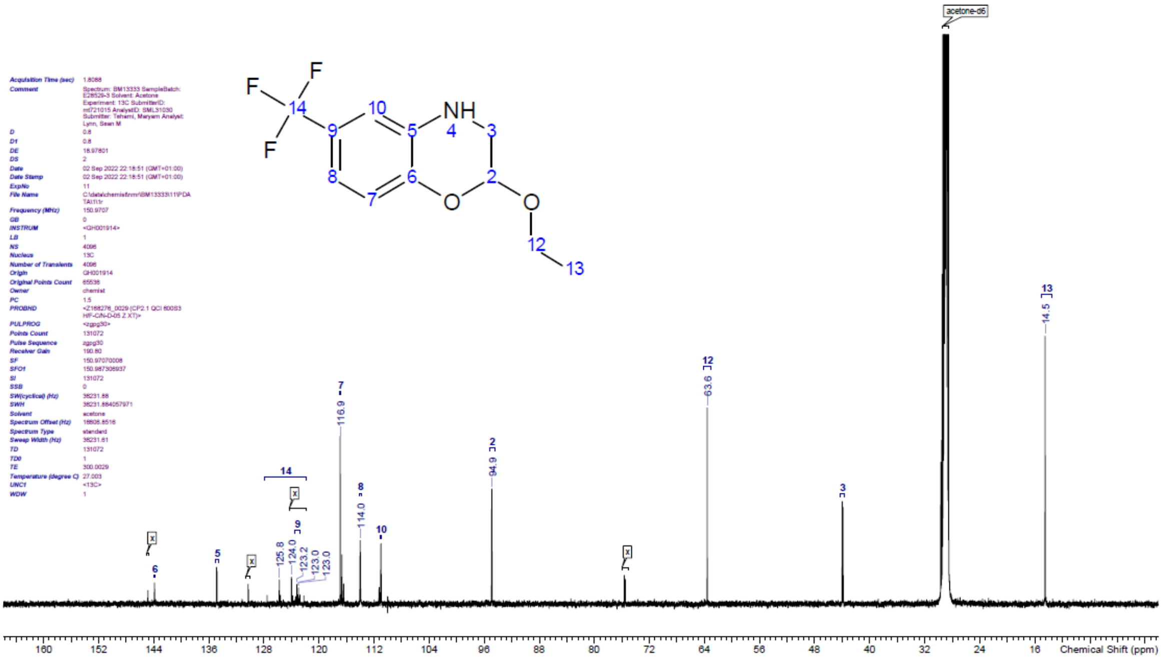

Figure S18. ^{13}C NMR Spectrum of **2B** in hexadeuteroacetone.

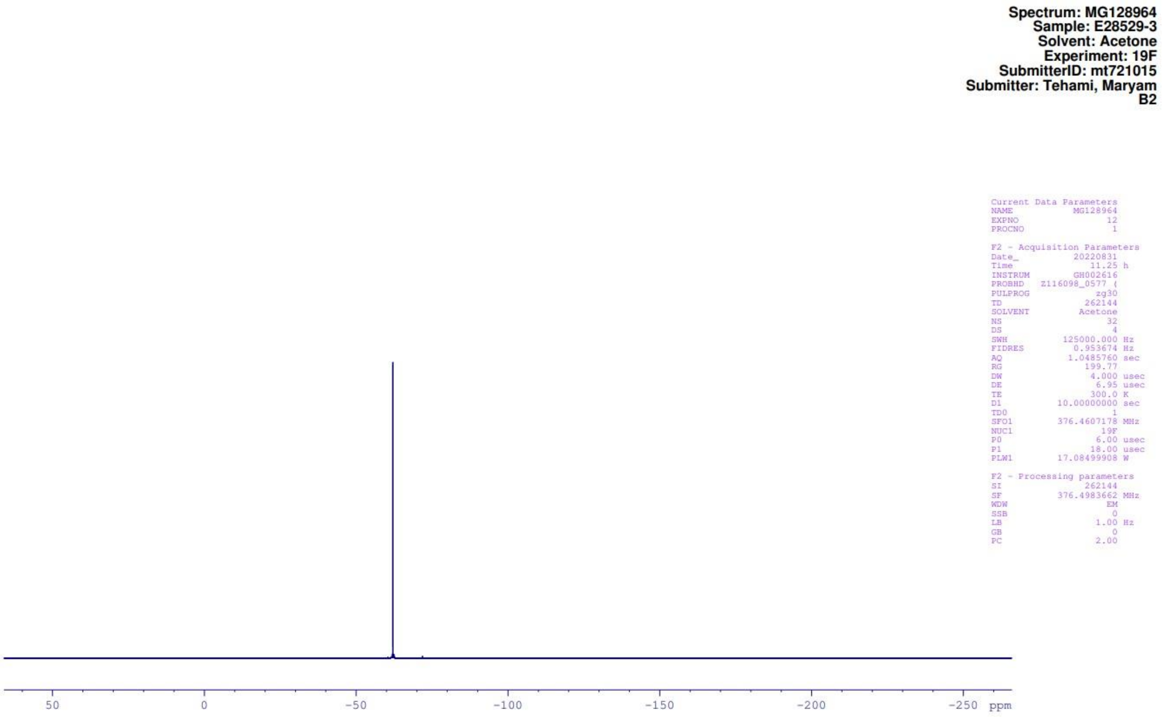

Figure S19. ^{19}F NMR Spectrum of **2B** in hexadeuteroacetone.

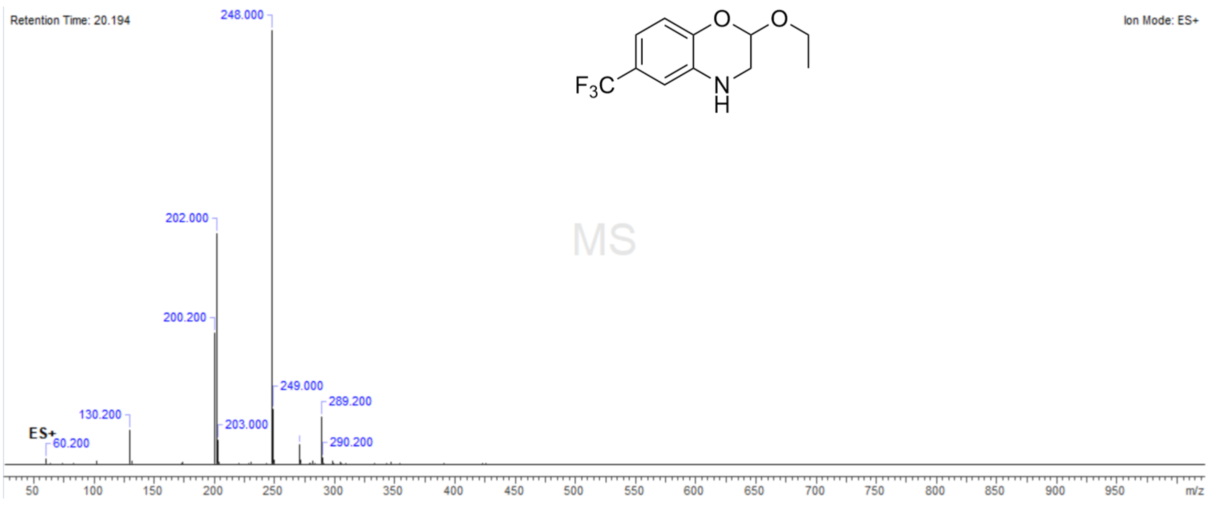

Figure S20. Positive mode electrospray mass spectrum of **2B.**

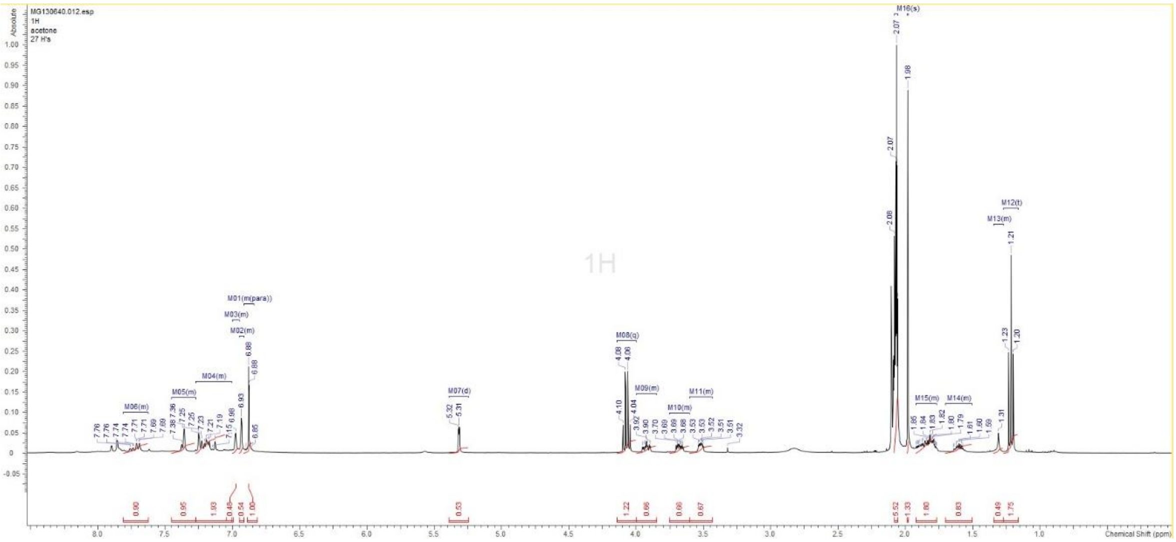

Figure S21. ^{1}H NMR Spectrum of **2C** in hexadeuteroacetone.

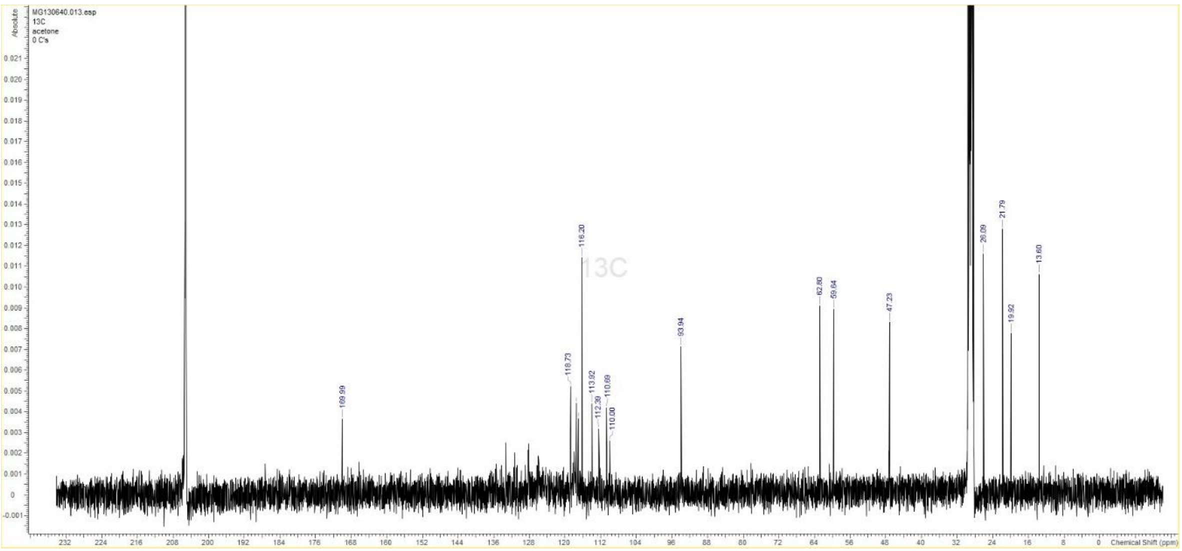

Figure S22. ^{13}C NMR Spectrum of **2C** in hexadeuteroacetone.

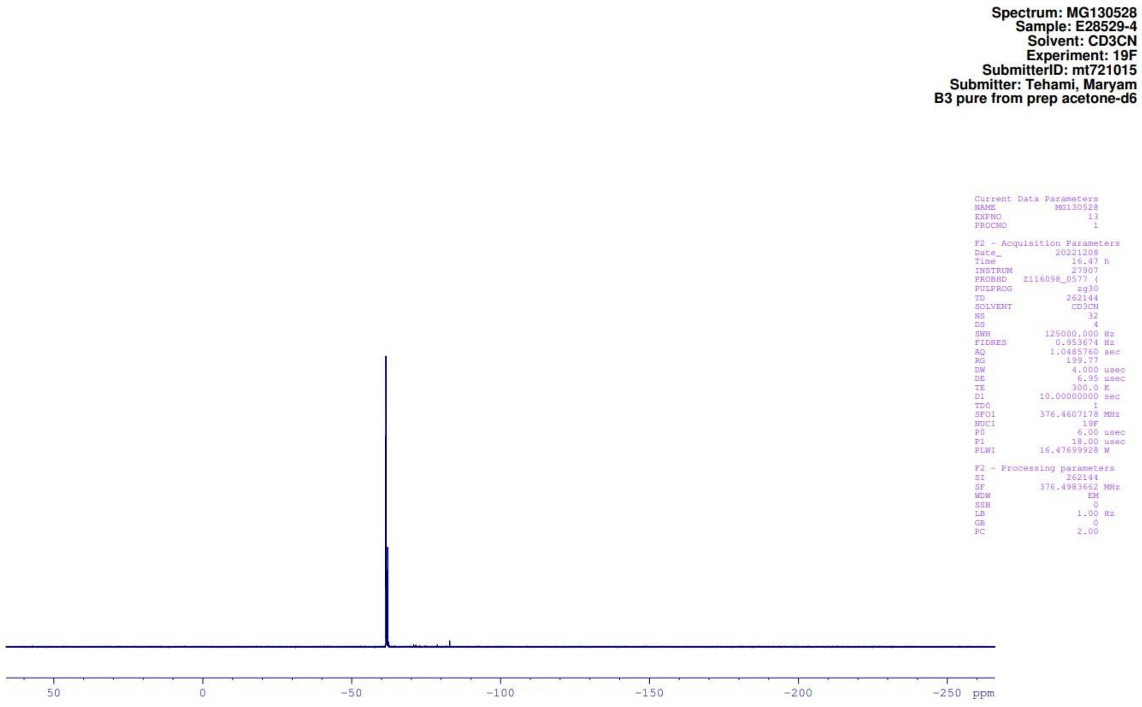

Figure S23. ^{19}F NMR Spectrum of **2C** in hexadeuteroacetone.

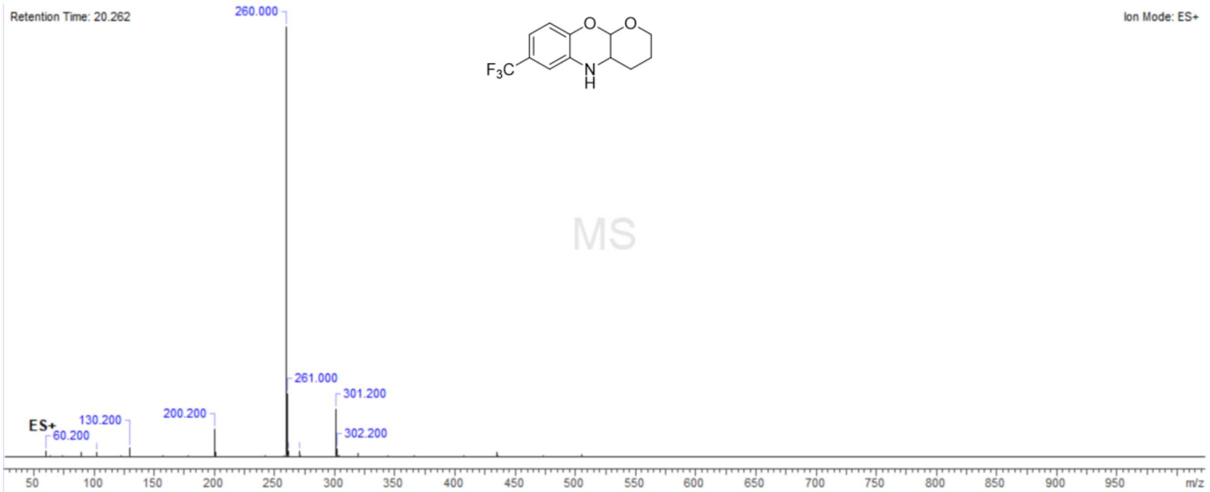

Figure S24. Positive mode electrospray mass spectrum of **2C.**

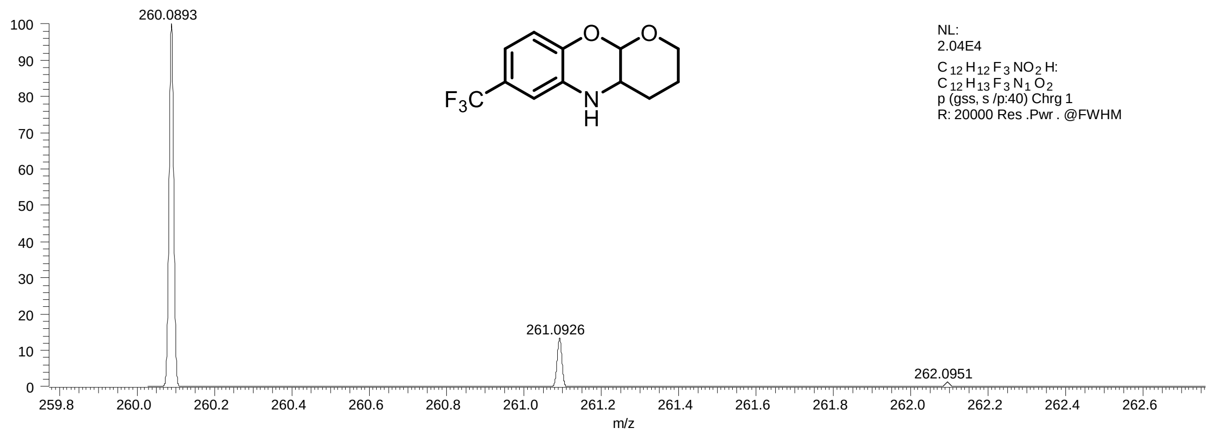

Figure S25. High Resolution Mass Spectrum of **2C.**

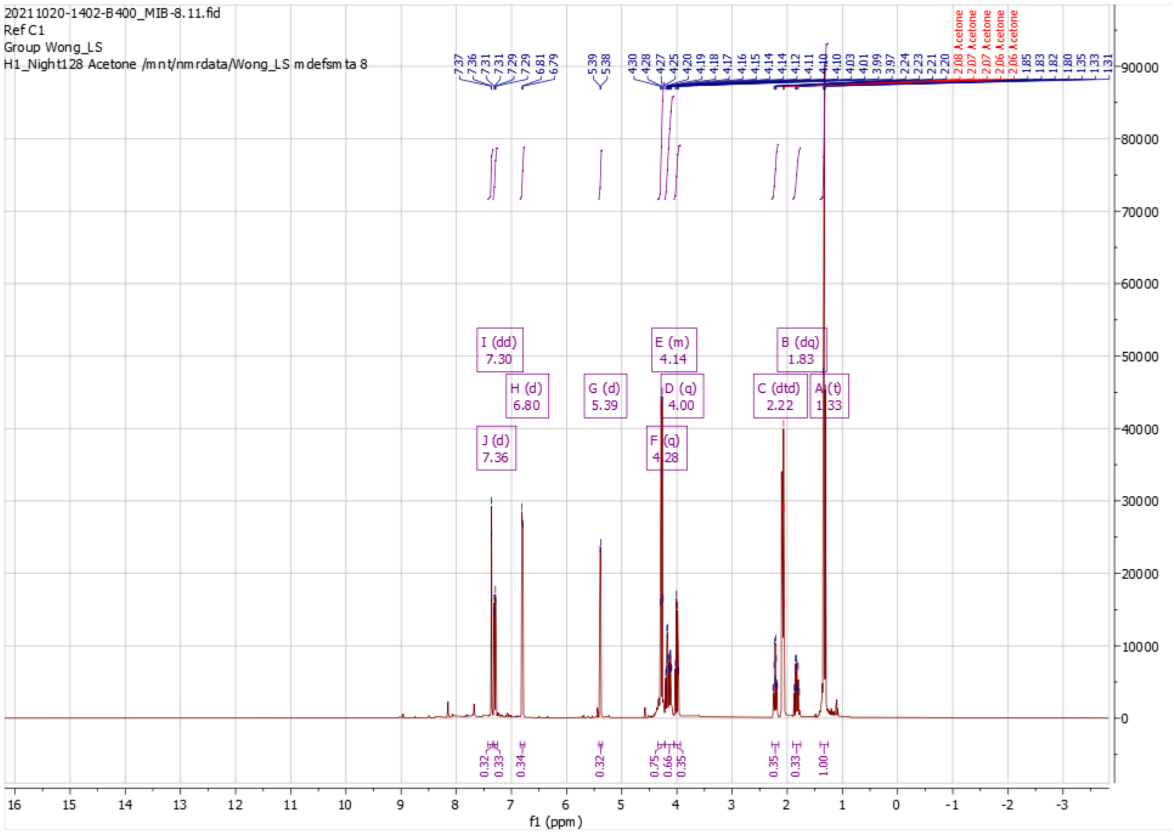

Figure S26. ^{1}H NMR Spectrum of **3A** in hexadeuteroacetone.

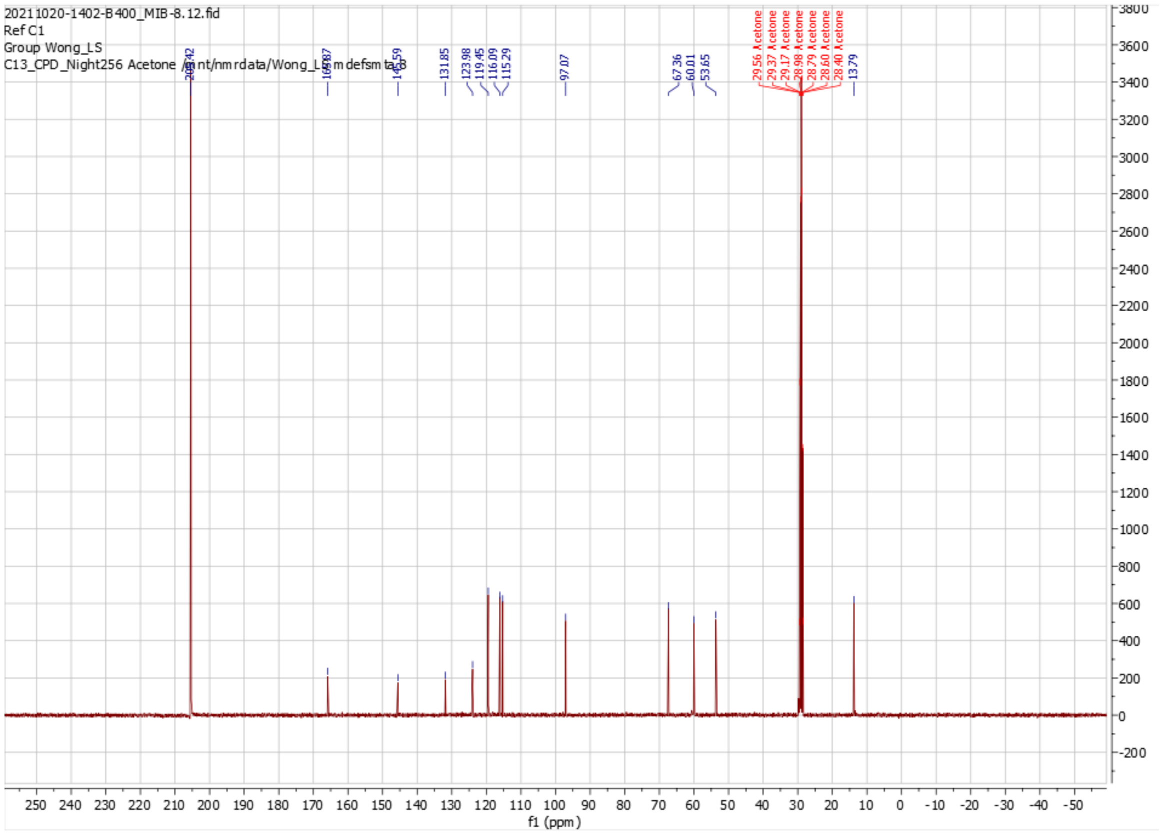

Figure S27. ^{13}C NMR Spectrum of **3A** in hexadeuteroacetone.

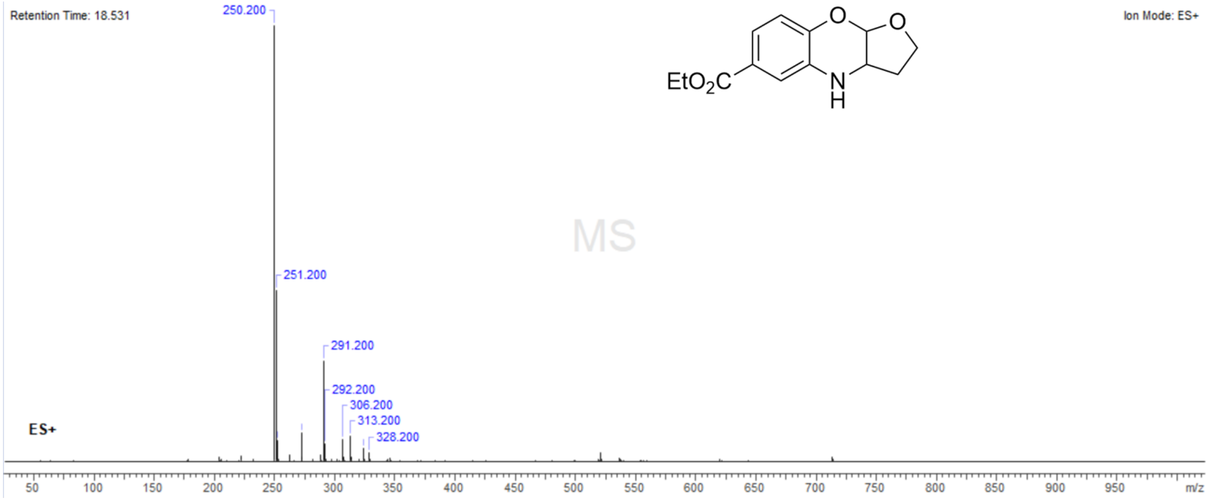

Figure S28. Positive mode electrospray mass spectrum of **3A.**

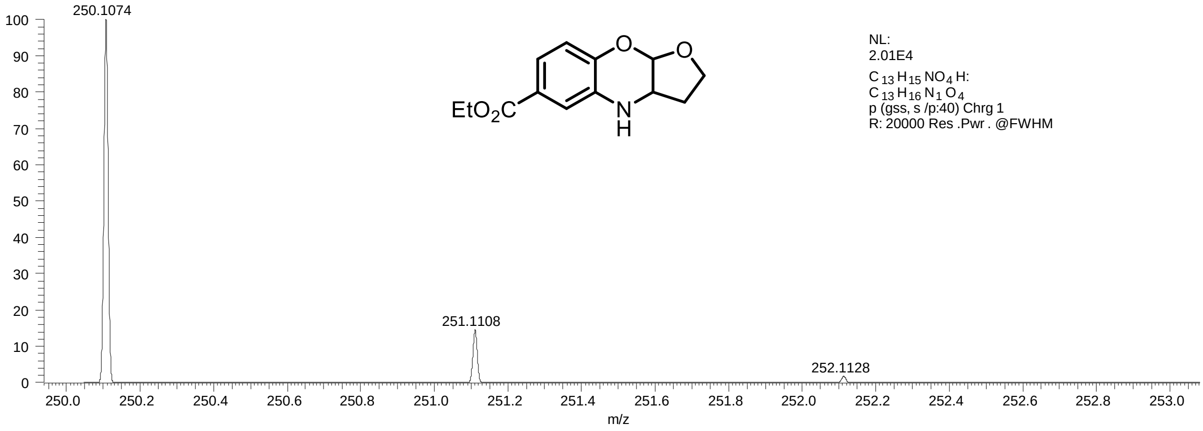

Figure S29. High Resolution Mass Spectrum of **3A.**

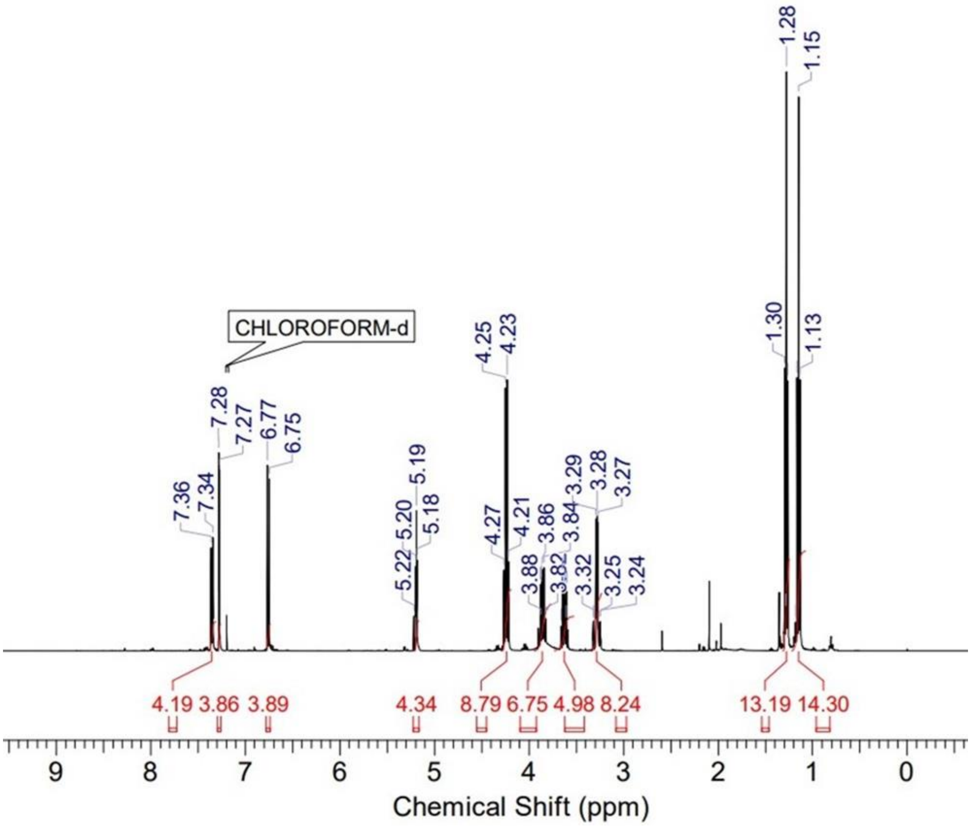

Figure S30. ^{1}H NMR Spectrum of **3B** in deuterochloroform.

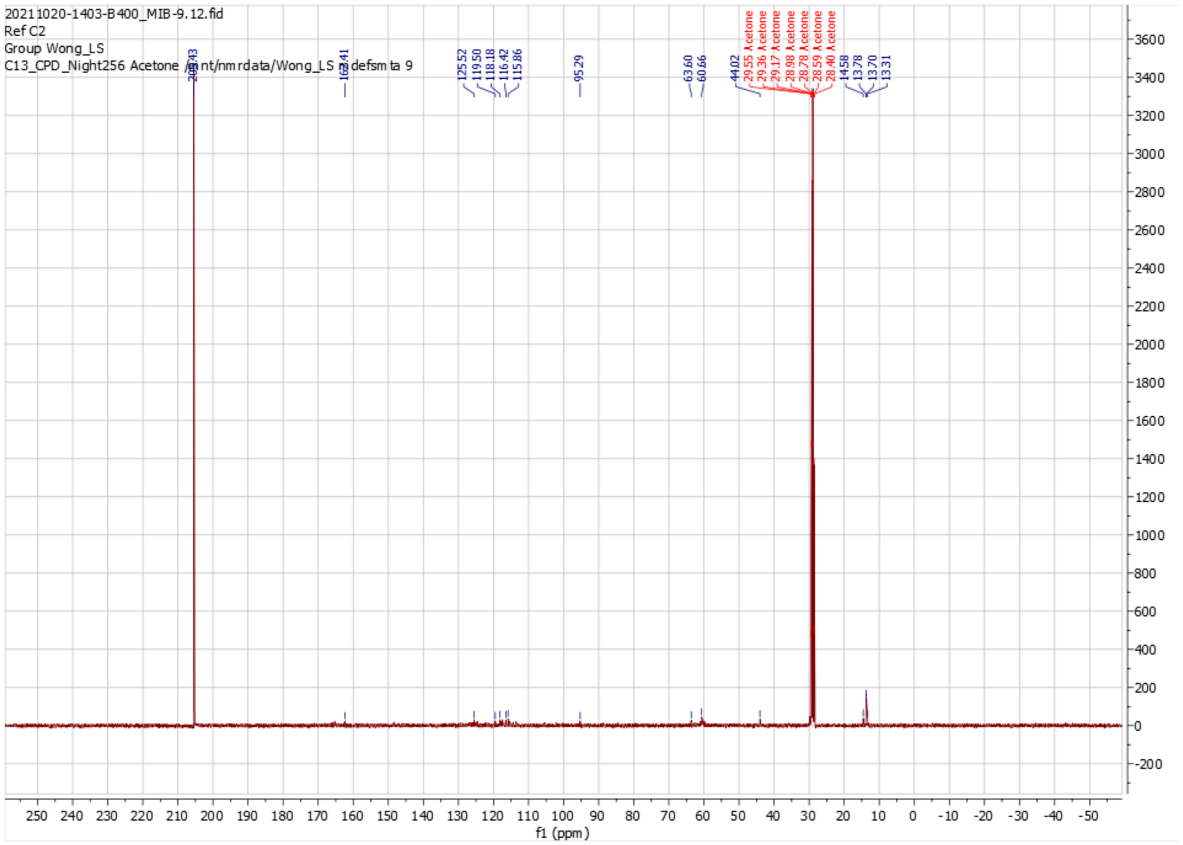

Figure S31. ^{13}C NMR Spectrum of **3B** in hexadeuteroacetone.

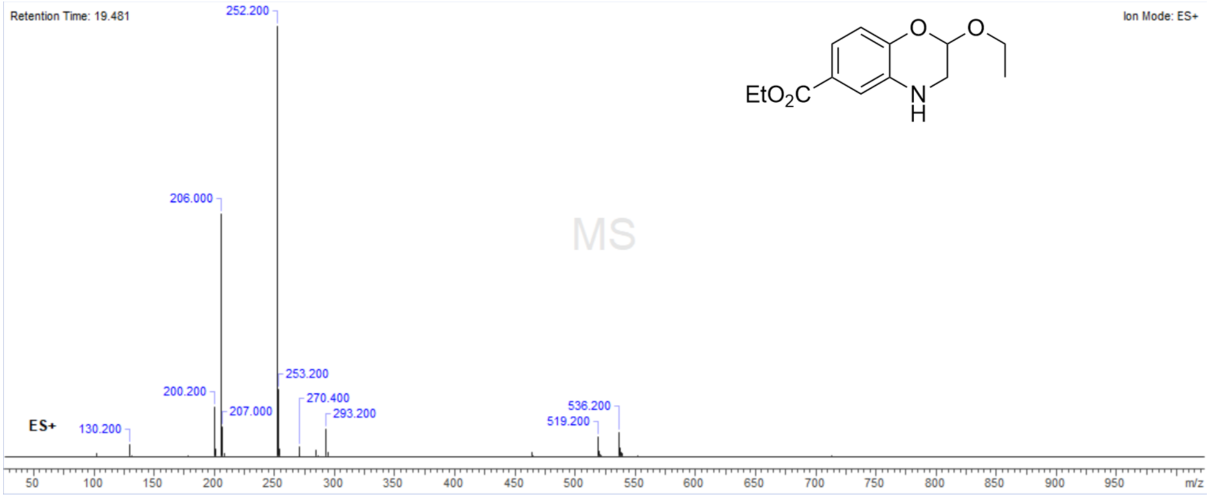

Figure S32. Positive mode electrospray mass spectrum of **3B.**

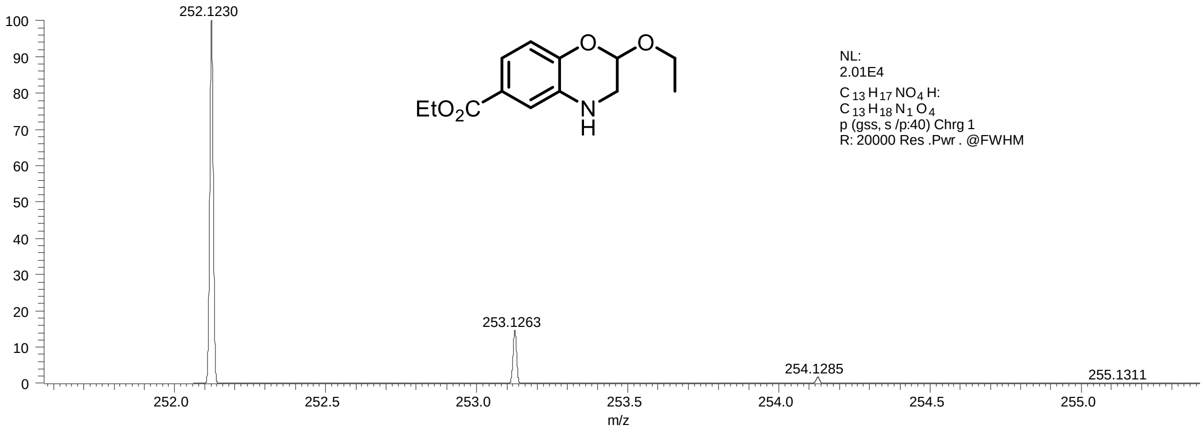

Figure S33. High Resolution Mass Spectrum of **3B.**

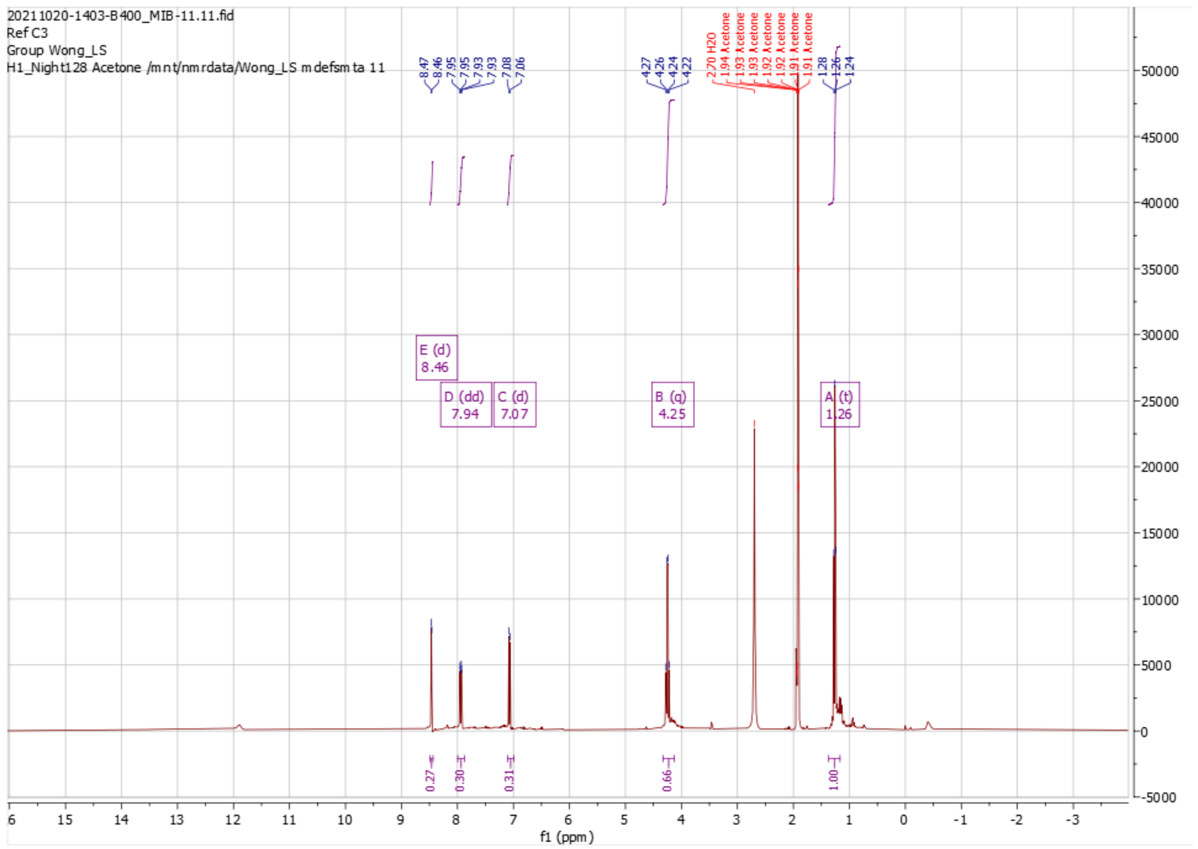

Figure S34. ^{1}H NMR Spectrum of **3C** in hexadeuteroacetone.

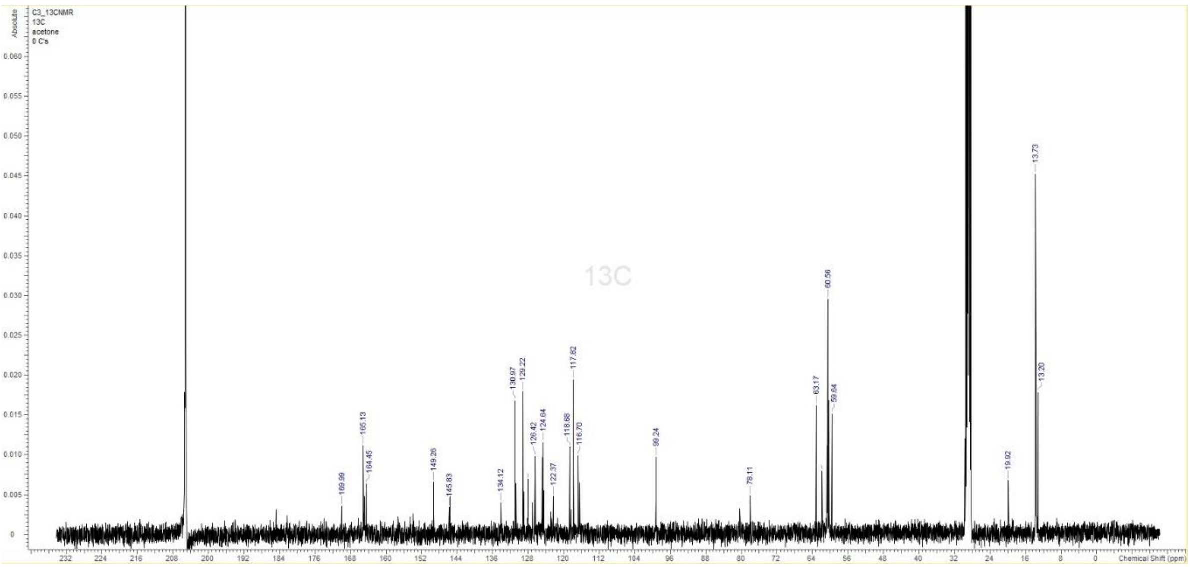

Figure S35. ^{13}C NMR Spectrum of **3C** in hexadeuteroacetone.

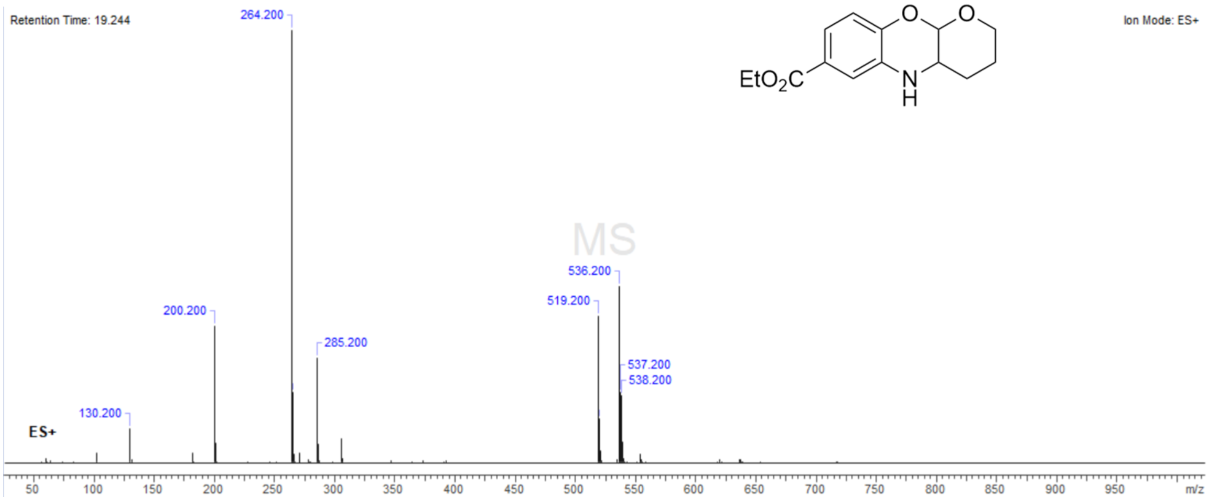

Figure S36. Positive mode electrospray mass spectrum of **3C.**

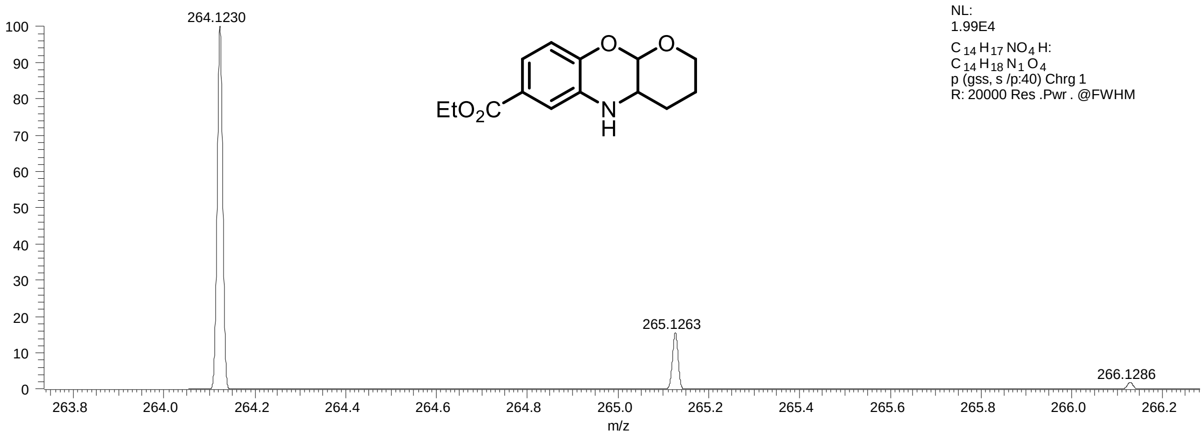

Figure S37. High Resolution Mass Spectrum of **3C.**

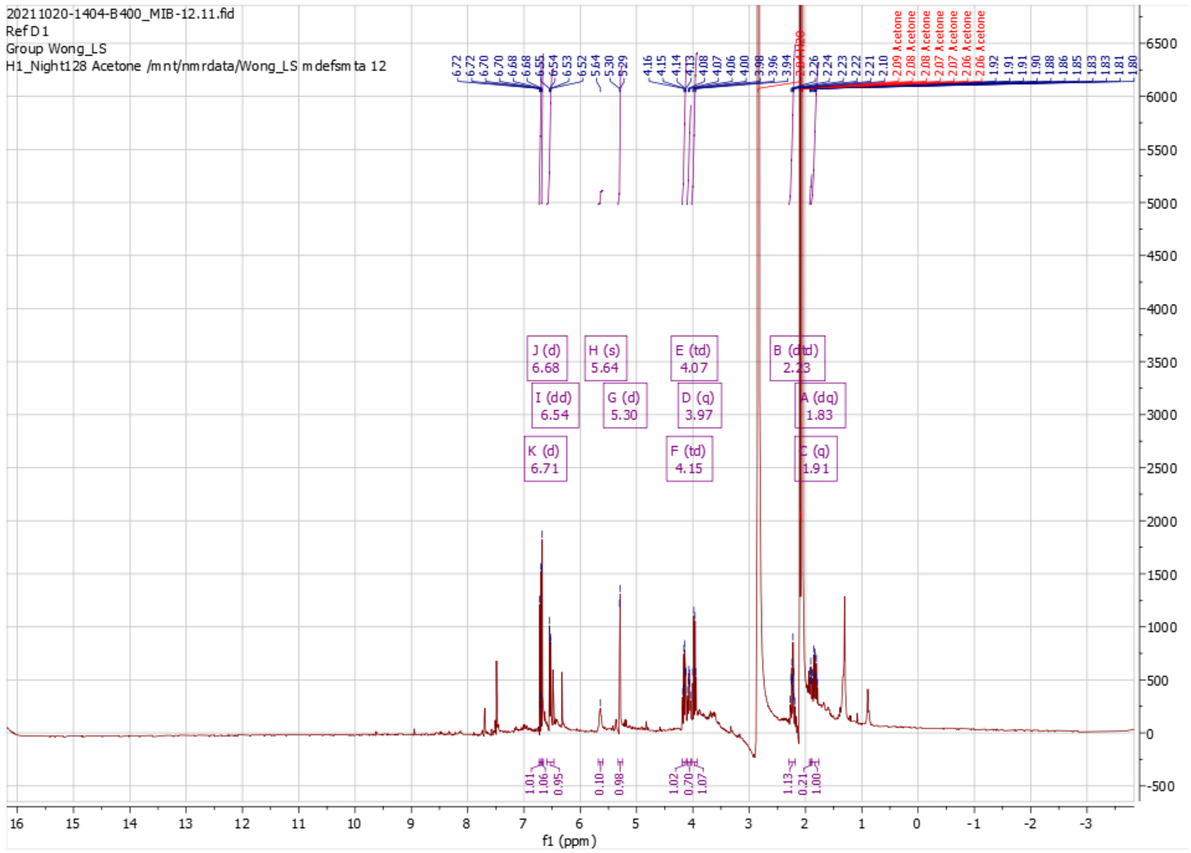

Figure S38. ^{1}H NMR Spectrum of **4A** in hexadeuteroacetone.

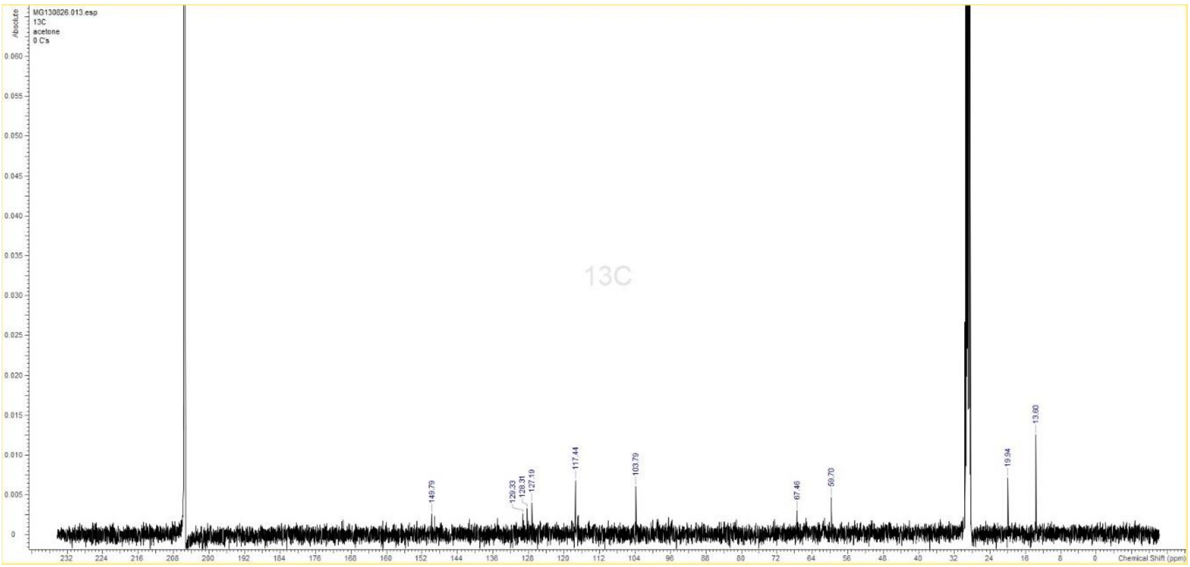

Figure S39. ^{13}C NMR Spectrum of **4A** in hexadeuteroacetone.

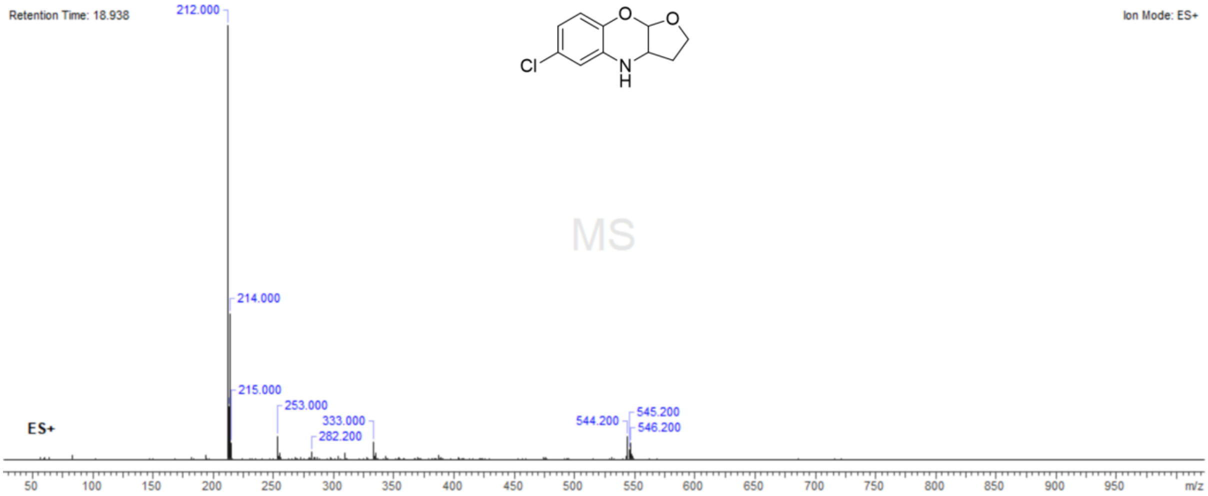

Figure S40. Positive mode electrospray mass spectrum of **4A.**

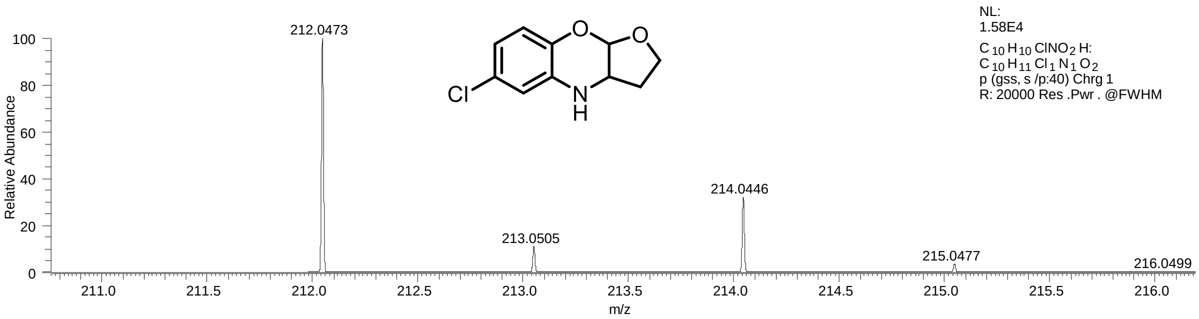

Figure S41. High Resolution Mass Spectrum of **4A.**
